# 20-Hydroxyecdysone, from Plant Extracts to Clinical Use: Therapeutic Potential for the Treatment of Neuromuscular, Cardio-Metabolic and Respiratory Diseases

**DOI:** 10.3390/biomedicines9050492

**Published:** 2021-04-29

**Authors:** Laurence Dinan, Waly Dioh, Stanislas Veillet, Rene Lafont

**Affiliations:** 1 Biophytis, Sorbonne Université, BC9, 4 place Jussieu, 75005 Paris, France; laurence.dinan@biophytis.com (L.D.); waly.dioh@biophytis.com (W.D.); stanislas.veillet@biophytis.com (S.V.); 2 BIOSIPE, IBPS, Sorbonne Université, UPMC, 75005 Paris, France

**Keywords:** anabolic, diabetes, Duchenne muscular dystrophy, β-ecdysone, ecdysteroid, ecdysterone, Mas1, osteoporosis, sarcopenia, COVID-19, cardiometabolic diseases, respiratory diseases

## Abstract

There is growing interest in the pharmaceutical and medical applications of 20-hydroxyecdysone (20E), a polyhydroxylated steroid which naturally occurs in low but very significant amounts in invertebrates, where it has hormonal roles, and in certain plant species, where it is believed to contribute to the deterrence of invertebrate predators. Studies in vivo and in vitro have revealed beneficial effects in mammals: anabolic, hypolipidemic, anti-diabetic, anti-inflammatory, hepatoprotective, etc. The possible mode of action in mammals has been determined recently, with the main mechanism involving the activation of the Mas1 receptor, a key component of the renin–angiotensin system, which would explain many of the pleiotropic effects observed in the different animal models. Processes have been developed to produce large amounts of pharmaceutical grade 20E, and regulatory preclinical studies have assessed its lack of toxicity. The effects of 20E have been evaluated in early stage clinical trials in healthy volunteers and in patients for the treatment of neuromuscular, cardio-metabolic or respiratory diseases. The prospects and limitations of developing 20E as a drug are discussed, including the requirement for a better evaluation of its safety and pharmacological profile and for developing a production process compliant with pharmaceutical standards.

## 1. Purpose

The aim of this review is to describe the potential and challenges in developing 20-hydroxyecdysone (20E) as a pharmaceutical agent for the future. It is a natural plant steroid with low mammalian toxicity and accumulated data from academic and pre-clinical studies showing that it possesses many beneficial pharmacological effects in mammals, including humans, but few of these properties have yet been substantiated by clinical trials. Further, the bioavailability of oral 20E is low. We shall describe and critically assess the approaches to the sourcing, purification, quality control, and assessment of the activity of the compound in mammalian systems and the clinical trials which are currently underway.

## 2. Ecdysteroids

Ecdysteroids are a family of invertebrate steroid hormones which are involved in the regulation of moulting, development and reproduction [1]. They differ significantly in their structure from vertebrate steroid hormones, since they are characteristically polyhydroxylated, retain the full C_8_ sterol side-chain, possess a 14α-hydroxy-7-en-6-one chromophoric group located in the B-ring and possess an A/B-*cis*-ring junction. Thus, they markedly differ from vertebrate steroid hormones in their polarity, bulk and shape, and there is no convincing evidence that ecdysteroids interact with nuclear receptors for the vertebrate steroids in mammals. It is generally accepted that 20E is the major biologically active form in insects, but other analogues act as biosynthetic intermediates (e.g., 2-deoxyecdysone), pro-hormones (ecdysone and/or 3-dehydroecdysone) or metabolites (e.g., 20,26-dihydroxyecdysone) or storage forms (e.g., ecdysteroid phosphates). Other ecdysteroids may be hormonally active in other invertebrates (e.g., ponasterone A in crustaceans). In accord with their hormonal role, the concentrations of ecdysteroids found in arthropods and other invertebrates are generally rather low (nM to μM), with the storage forms being present in the highest amounts where they occur.

**Figure 1:**
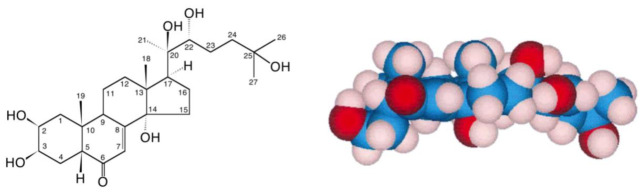
20-hydroxyecdysone (20E; β-ecdysone; crustecdysone; ecdysterone; BIO101; CAS 5289-74-7; IUPAC 2β,3β,14α,20*R*,22*R*,25-hexahydroxy-5β-cholest-7-en-6-one).

In addition to ecdysteroids occurring in invertebrates (zooecdysteroids), they are also present in certain plant species as phytoecdysteroids, where they are believed to contribute to the deterrence of invertebrate predators. They are present in detectable amounts in the seeds of 5–6% of investigated plant species, and in leaves of an even greater proportion of species [2]. Concentrations vary from just detectable to high, depending on the species, the plant part and the stage of development, accounting for 1–2% of the dry weight in high accumulators. Phytoecdysteroid profiles may vary from simple (the presence of one or two major components), through intermediate (a mixture of major and minor components) to complex (a cocktail of many analogues) [3]. 20E (Figure 1) is the most frequently encountered phytoecdysteroid and very frequently is the major phytoecdysteroid present in the plant. Currently, 526 natural ecdysteroid analogues have been identified (Ecdybase [4]), most of which have been isolated only from plants, probably in large part because of the higher concentrations found in plant sources; some are found in invertebrates and plants, and a few have only so far been detected in invertebrates. To date, the only ecdysteroid which is commercially available at a reasonable cost and in large (kg) amounts is 20E (Figure 1), which can be isolated efficiently from specific high accumulating species (see later).

## 3. A Traditional Medicinal Product

Research in this area has a long history and started soon after the structural identification of ecdysone (E) and 20-hydroxyecdysone (20E) from arthropods and the discovery of analogues in plants in 1965 [5], when it was thought that the compounds might be useful invertebrate control agents. As a consequence, considerable effort was put into the total chemical synthesis of E and 20E (reviewed in [6]), determining the toxicity and initial pharmacokinetic studies in mammals. Synthesis proved possible but inefficient, and the physico-chemical properties (polarity, UV stability, etc.) were not really compatible with field application. Western scientists tended to focus on the metabolism and mode of action of ecdysteroids within invertebrates, while Eastern European and Central Asian scientists continued to explore the pharmaceutical uses of ecdysteroids. The findings of these latter studies were published in Slavic and Turkic languages, so their significance was not fully appreciated for quite some time [7]. In the past two decades, there has been a considerable increase in studies focusing on the effects and potential applications around the world, which has been enabled by the concommitant commercial availability of larger amounts of pure 20E, such that it is now fair to say that this is currently the major focus of ecdysteroid research, leaving invertebrate ecdysteroid research rather in the shade.

As the phytochemical analysis of traditional medicinal plants from around the world has advanced, it has become apparent that some of them contain significant amounts of ecdysteroids (Table 1). This interesting association may indicate that ecdysteroids, alone or in conjunction with other plant secondary compounds, may have very wide therepeutic applications, but it must be borne in mind that in very few of these cases have the ecdysteroids been proven to be responsible for the pharmacological activity.

## 4. Pharmacologial Effects of 20E in Animals

Table 2 provides a summary of the effects attributed to 20E in mammals. This topic has been extensively reviewed previously [7,33,34,35,36,37,38,39,40,41,42,43,44,45], so here we shall just present some data from more recent studies on the anabolic and hypolipidemic effects by way of example.

## 5. Protein Synthesis Stimulatory Effect In Vitro

**Figure 2:**
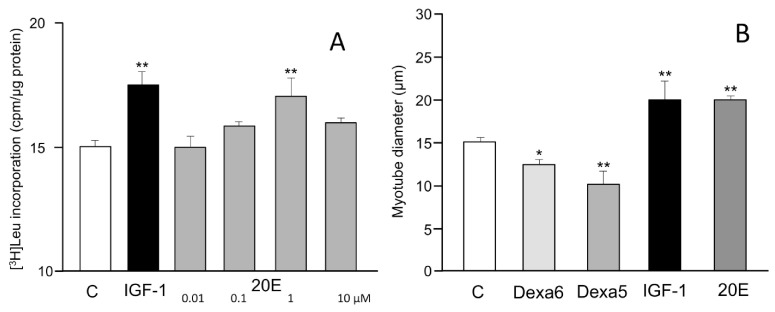
(**A**) The effects of 20E (0.01–10 μM) on protein synthesis in C2C12 cells, showing the anabolic effect of 20E on optimal value for 1 µM 20E. C2C12 cells were grown on 24-well plates at a density of 30,000 cells/well in 0.5 mL of growth medium (Dulbecco’s Modified Eagle Medium (DMEM) 4.5 g/L glucose supplemented with 10% fetal bovine serum). Twenty-four hours after plating, the differentiation induction into multinucleated myotubes was carried out, and after 5 days, cells were pre-incubated in Krebs medium 1 h at 37 °C before being incubated in DMEM media without serum for 2.5 h in the presence of [^3^H]-Leucine (5 µCi/mL) and DMSO (control condition) or IGF-1 (100 ng/mL) or 20E (0.01–0.1–1–10 µM). At the end of incubation, supernatants were discarded and cells were lysed in 0.1 N NaOH for 30 min. The cell soluble fraction-associated radioactivity was then counted and protein was quantified using the Lowry method (after [103]). (**B**) Effects of dexamethasone (Dexa 6 = 10^−6^ M, Dexa 5 = 10^−5^ M), IGF-1 (10 ng/mL), and 20E (10^−6^ M) on the diameter of C2C12 myotubes. Four- to six-day-old myotubes were incubated for 48 h with test chemicals, and were fixed and photographed by glutaraldehyde-induced autofluorescence. *: *p* = < 0.05; **: *p* = 0.01. (redrawn and modified from [54]).

The murine C2C12 cell line is an accepted model system for myotubule formation and action and consequently for the investigation of the activity of anabolic and anti-sarcopenic agents. The cells in culture can be induced to differentiate into myotubules and then be treated with the test agents. The incorporation of radioactive leucine into protein is a measure of the anabolic activity of the test compound. Figure 2A illustrates the dose–response protein synthesis stimulatory activity of 20E, which is equally active as insulin-like growth factor-1 (IGF-1) in this system. Alternatively, the anabolic effects can be evaluated by measuring myotube diameters. Similar results were observed, e.g., by [49,54] (Figure 2B).

**Figure 3:**
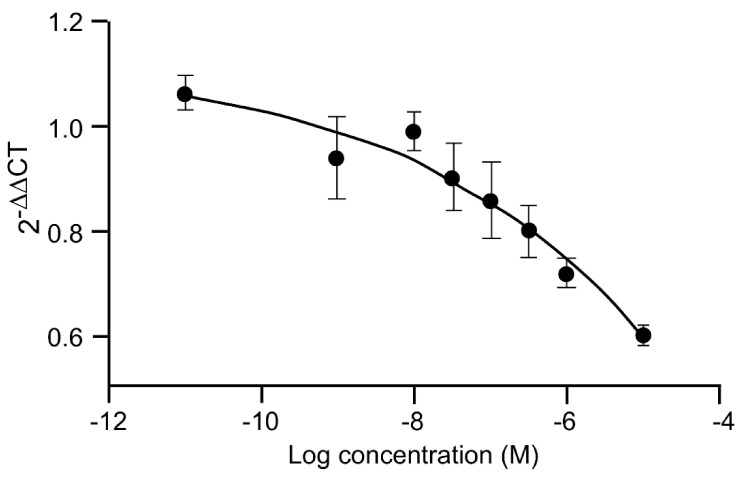
Dose-dependent inhibition of myostatin gene expression in C2C12 cells by 20E. C2C12 mouse myoblasts were differentiated for 6 days into myotubes. They were then treated for 6 h with concentrations of 20E ranging from 0.001 to 10 μM. Myostatin gene expression was detected by qRT-PCR. Results are shown as means ± standard error of the mean (SEM) ([103]).

Myotube growth in C2C12 cells is mediated negatively by myostatin, and IGF-1 markedly reduces myostatin gene expression (Figure 3). 20E mimics the effect of IGF-1 in a dose-dependent manner, with significant difference from the control occurring at 1–10 μM. Such an effect was also observed by [53].

## 6. Anti-Obesity Effect

**Figure 4:**
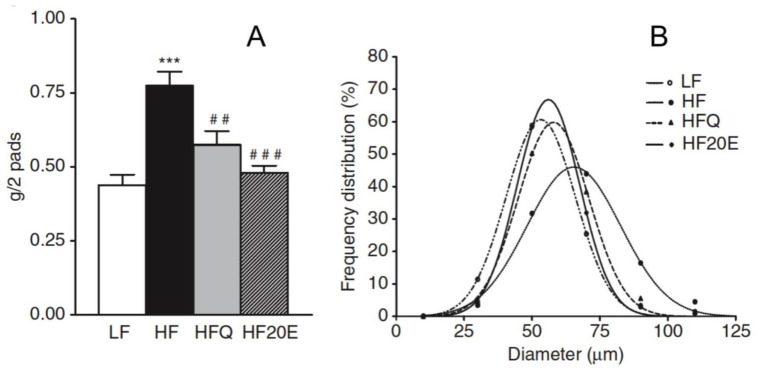
Effect of 20E on mice fed a high-fat diet (HF), when compared to mice fed a low-fat diet (LF). The animals received either pure 20E (50 mg/kg/day) or the same amount of 20E as a quinoa extract (Q). Panel (**A**) shows the impact on the mass of epididymal adipose tissue (*** *p* < 0.01 when compared to LF; ## *p* < 0.01 and ### *p* < 0.001 when compared to HF) and panel (**B**) shows the effect on adipocyte diameter (reproduced, with permission, from [59]).

The effects of 20E on adiposity are well documented (Table 2). Mice fed on a high-fat diet tend to adiposity and this is reflected in the mass of epididymal fat pads and the diameter of the adipocytes. Figure 4 compares these parameters in mice on low-fat, high-fat and high-fat +20E diets. 20E counteracts both the increase in the mass of the fat pads and retains the median adipocyte diameter close to that in mice fed a low-fat diet. Similar findings have been reported by other authors, e.g., [49]. 20E is also efficient in high-carbohydrate (high fructose) fed gerbils [61].

## 7. Mechanism of Action of 20E in Mammals

Early studies showed that 20E displays pleiotropic effects on mammals [104]:

- Increases protein synthesis in skeletal muscles and heart [46,105]
- Increases ATP synthesis in muscles [106]
- Stimulates production of erythrocytes [74]
- Decreases hyperglycemia in diabetic animals [63]
- Reduces plasma cholesterol levels [107]
- Decreases the activity of triglyceride lipase [108]
- Protects against experimental atherosclerosis in rabbits [109]
- Activates acetylcholinesterase in the brain [110]
- Activates glutamate decarboxylase (=GABA synthesis) in the brain [111]
- Possesses immunomodulatory activity [112].

In view of these diverse effects, it could be expected that 20E might have various modes of action at the cellular level. There are no homologues to the arthropod ecdysteroid receptor (EcR) in mammals, and attempts to show high affinity binding of ecdysteroids to vertebrate nuclear receptors have not been successful [88,113], but see the possible involvement of estrogen receptor-β (ERβ) below. The mode(s) of action of 20E in mammals has not been fully elucidated for any responsive cell type. In part, this is because the action appears to be non-genomic and also because relatively high concentrations (0.1–10 μM) of 20E are required to observe effects, implying relatively low affinity interaction of 20E with its cellular target(s).

**Figure 5:**
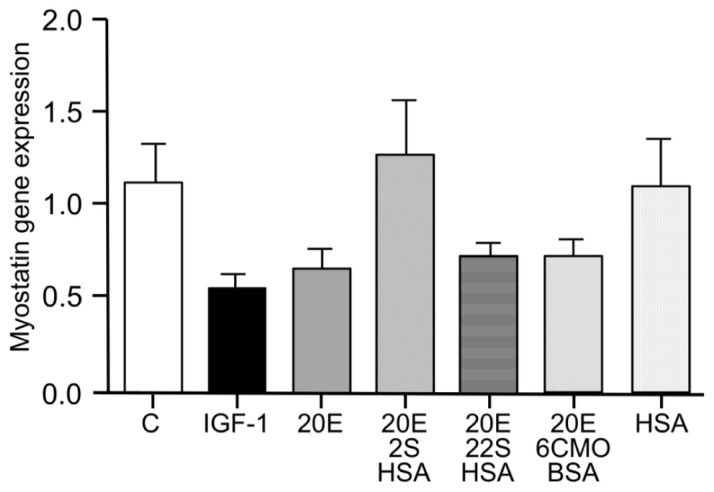
20E reduction of myostatin gene expression in C2C12 cells (differentiated for 6 days into myotubules) is mediated by binding to receptor sites on the external surface of the cells. The histogram compares the activities of IGF-1 (100 nM) and 20E (10 μM) with those of conjugates of 20E covalently bound to protein (HSA or BSA) through different C-atoms (C-2 and C-22-hemisuccinates or C-6 [6-carboxymethoxime]), all at nominal 10 μM hapten concentration. BSA bovine serum albumin; HSA: human serum albumin; error bars = standard error of the mean [103,114].

Several models for the modes of action of 20E at responding mammalian cells are being pursued. The models are not mutually exclusive, and it is possible that they could operate simultaneously or in different tissues. Given the pleiotropic effects described for 20E on mammalian cells, it seems possible that it operates through several signalling pathways, which could be more or less tissue-specific. Gorelick-Feldman et al. [50] clearly established that 20E acts through a membrane GPCR receptor, and this has been confirmed using albumin-bound 20E, which cannot cross cell membranes (Figure 5).

The renin–angiotensin system (RAS) is strongly implicated in maintaining muscle function, and one of the peptide products of this system, angiotensin II, targets skeletal muscle cells via the AT1R receptor and has been implicated in the development of sarcopenia [115], both directly through AT1R, which results in increased resistance to insulin and IGF-1, and indirectly by increased production of myostatin, glucocorticoids, TNF-α, and IL-6. Consequently, ACE inhibitors (which inhibit the production of angiotensin II) have found some application in the treatment of sarcopenia [116]. Another component of the RAS is angiotensin 1-7, which has been identified as the natural ligand for another GPCR, Mas1, the activation of which has been hypothesized to enhance protein synthesis in muscle cells. Thus, RAS would have a “harmful arm” acting through AT1R where the activation of the receptor enhances proteolysis, but this can be counteracted by a “protective arm”, acting through Mas1, where the activation of the receptor enhances protein synthesis. According to this hypothesis, protection against muscle wasting can be achieved by reducing the activation of AT1R, or the activation of Mas1, or a combination of the two. There are reasons to believe that the activation of Mas1 would be more effective at stimulating muscle anabolism and reducing adipose tissue than an ATIR antagonist, and it would have fewer side-effects than ACE inhibitors. The Mas1 receptor is expressed in many tissues, and its activation in different tissues (e.g., heart, kidney, CNS) may evoke various protective effects [117]. The activation of Mas1 by 20E could explain the anabolic effects of 20E on muscle cells [103,113,114].

Angiotensin(1-7) partially inhibits myostatin gene expression and this inhibition is abolished by the angiotensin(1-7) antagonists D-Pro^7^-Ang(1-7) and D-Ala^7^-Ang(1-7) (A779). These antagonists are also effective at preventing the inhibition brought about by 20E, indicating that Ang(1-7) and 20E operate through Mas1 activation [113]. The gene interference of the Mas1 receptor using silencing RNA (siRNAMas1) provided further evidence for the involvement of Mas1 in the mode of action of 20E: siRNAMas1 was found to reverse the inhibition in myostatin gene expression brought about by Ang(1-7) or 20E [113].

Parr et al. [54] have put forward an alternative model for the mode of action of 20E in bringing about the hypertrophy of mammalian skeletal muscle cells, which involves the interaction of the steroid with nuclear estrogen receptor-β (ERβ). Using C2C12 cells, they observed that both 20E (1 μM) and E2 (1 nM) enhanced myotube diameter. Co-treatment with the anti-estrogen ZK191703 (ZK) antagonized the hypertrophy brought about not only by E2, but also by 20E, indicating that E2 and 20E share a common mode of action. The authors next showed that a reporter gene under the control of an estrogen response element could be activated dose dependently by E2 or 20E interacting with ERα or Erβ, and that this activation could be prevented if ZK was also present. Use of the estrogen receptor-specific agonists, ALPHA (for ERα) and BETA (for ERβ), indicated that ERβ mediated the hypertrophy of the C2C12 myotubes, but a note of caution is required because these agonists are only selective at low concentrations and the BETA dose–response curve was bell-shaped. Finally, the selective ERβ-antagonist (ANTIBETA) antagonized the effects of E2 and 20E in the C2C12 cells. The authors suggest that, as it had previously been shown that ERβ can modulate Akt phosphorylation, this could be the link by which 20E brings about hypertrophy in the C2C12 cells. In silico docking studies suggest that 20E can interact with the ligand-binding domains of ERα and ERβ but has the potential for stronger interaction with ERβ [118]. However, the exact nature of the receptor involved in the observed effects can be questioned, because all binding studies performed with nuclear receptors ERα and ERβ were negative [e.g., 38], and truncated membrane-bound forms of ER receptors have been described that may display different ligand specificity [119]. Finally, the recent article by Sobrino et al. [120] provides evidence that in primary human umbilical vein endothelial cells cultures, E2 acts via a membrane-bound ER to bring about NO-dependent vasodilation, and they show that this effect also requires Mas activation as it is abolished by a Mas inhibitor. Although this is a different system to the muscle cells above, it might perhaps give an indication of how both 20E and E2 can bring about muscle hypertrophy.

**Figure 6:**
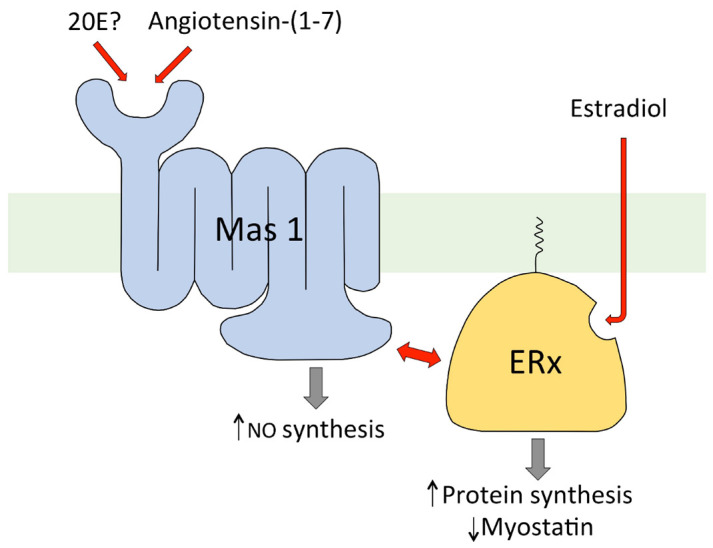
Diagrammatic representation of the proposed mode of action of 20E in the regulation of protein synthesis in C2C12 muscle cells in vitro ([113]).

Further experiments with C2C12 cells [113] indicate that the two hypotheses for the mode of action of 20E on these cells might be unified. Estradiol (E2) was found to inhibit myostatin gene expression in a dose-dependent manner (0.1–10 μM). To determine if the action of E2 is dependent on interaction with a membrane receptor, the activity of E2 was compared with that of E2-CMO (estradiol 6-carboxymethyloxime) and E2-CMO-BSA (E2-CMO covalently coupled to BSA). E2-CMO was found to inhibit myostatin gene expression in C2C12 cells like E2, showing that the carboxymethyl oxime derivative retained biological activity. In contrast, the E2-CMO-BSA conjugate was inactive, so it appears that E2 must enter the cell to be active. In addition to the classical nuclear receptors (ERα and ERβ), several non-nuclear forms of ER are known, and it is presumably one of these which is involved in the regulation of myostatin gene expression in C2C12 cells. Additionally, 17-*epi*-estradiol (17αE2), a close analogue of E2 which does not bind the nuclear receptors ERα and ERβ, was found to inhibit myostatin gene expression in C2C12 cells, providing further evidence for the involvement of a non-nuclear ER [113]. By combining the available experimental data, it has been possible to put together a hypothesis for how both 20E and E2 can regulate myostatin gene expression, whereby 20E interacts externally to the cell with the integral membrane Mas1 receptor, which in turn interacts with ERx located on the inner side of the plasma membrane, while E2 enters the cell to interact with ERx, bringing about myostatin regulation in a more direct way (Figure 6).

This finding may have important consequences because the Mas receptor is expressed in many tissues, which fits with the large array of physiological effects of its endogenous ligand Ang(1-7), which are also observed for 20E [113].

## 8. Toxicity, Bioavailability, Pharmacokinetics and Metabolism in Animal Models

### Toxicity

Ogawa et al. [121] determined LD_50_s for ingested 20E or inokosterone in mice of >9 g/kg and LD_50_s of 6.4 g/kg and 7.8 g/kg for i.p.-injected 20E and inokosterone, respectively. Thus, ecdysteroids are regarded as non-toxic to mammals. Similarly, no subacute toxicity was observed in long-term feeding experiments (0.2–2 g/kg/day) in rats. Additionally, no effects were seen after the administration of these two ecdysteroids to bullfrogs (up to 600 mg/kg in the lymph sinus) or rabbits (up to 100 mg/kg). The two ecdysteroids did not have sex hormonal activity or, interestingly in the context of more recent studies, anti-inflammatory or anabolic effects in rats. Additionally, rats and birds can feed on seeds of *Leuzea carthamoides*, which contain 2% (*w*/*w*) ecdysteroids (mainly 20E), and thrive [7].

Additionally, Seidlova-Wuttke et al. [57,94] fed ovariectomized rats with up to 500 mg/kg daily for 3 months with no stated detrimental effects, but no data on survival are presented in the articles. More recently, a purified to pharmaceutical grade 20E (≥97% 20E purity) was used as a drug candidate in preclinical regulatory studies including safety pharmacology, genotoxicity and repeated toxicology in rodents (rats) and non-rodents (dog). This drug candidate depicted a good safety profile with no genotoxic effects and No Observed Adverse Effect Levels (NOAEL) established at 1000 mg/kg in repeated dose toxicity studies in rats (26-week) and dogs (39-week) (Biophytis, unpublished data).

## 9. Pharmacokinetics and Metabolism in Mice—Early Studies

The earliest study on the fate of radiolabelled 20E in a mammalian species was performed on mice [122] and showed after oral administration that the bioavailability was low and that elimination was essentially fecal, while after i.p. administration, a somewhat higher level was found in the urine, even though fecal excretion still predominated. Of the radioactivity crossing the intestinal tract after oral administration (<2% of that administered), most was associated with the liver and bile duct, providing the first evidence for an entero-hepatic cycle for ingested ecdysteroids.

Lafont et al. [123] studied the metabolism of ecdysone, the only commercially available radioactive molecule when the study was made, in mice after intraperitoneal injections of the radioactive molecule. Ecdysone levels increased transiently in the liver and then accumulated in the intestine, which, within one or two hours after injection, contained almost all the radioactivity. One day after injection, most of the radioactivity had been eliminated from the body. Moreover, the metabolism of 20E was faster after intraperitoneal injection than after ingestion [122]. As with ecdysone, and whatever the mode of administration, radioactivity was rapidly taken up by the liver and then excreted into the intestine via the bile [122]. Excretion was again primarily fecal in mice [123]. The distribution of radioactivity from ^3^H-labelled 20E after injection into the caudal vein of mice has been studied by Wu et al. [124]. The kinetics of distribution showed a rapid elimination in urine and an uptake by the liver and bile, then most radioactivity accumulated into the intestine lumen before being eliminated in feces. Unfortunately, no information was provided about the associated metabolism [124].

Studies on the metabolism radiolabelled ecdysone in i.p.-injected mice [123,125] showed that the radioactivity was excreted fully within 24h unless it was diluted with unlabelled ecdysone, when the excretion of radioactivity was extended over 3 days. The injected radioactivity was rapidly captured by the liver and transferred to the intestine, where essentially all of it was located within 45–60 min. The principal metabolites formed were single or multiple products of 14-dehydroxylation, reduction of the 6-oxo group (6α-OH), reduction of the 7,8-double bond and 3-epimerisation. It is significant that ecdysone does not undergo side-chain cleavage because it does not possess a 20,22-diol and cannot be readily hydroxylated at C-20 after injection or ingestion in mice.

## 10. Pharmacokinetics after Oral Administration in Mice—More Recent Studies

**Figure 7:**
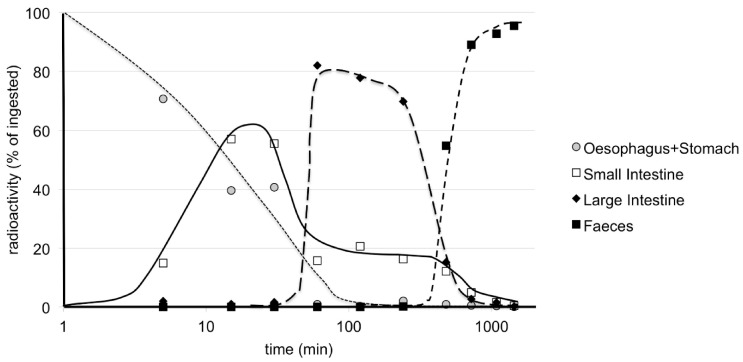
Time-course of the distribution of radioactivity in the stomach/intestine/feces in mice after the oral application of [1α,2α-^3^H_2_]20E. Note the logarithmic scale for abscissa. Each value is a mean of 2 animals. Note the plateau of small intestine content that is best explained by an entero-hepatic cycle and consistent with the prolonged concentration of radioactivity in bile as observed by Wu et al. [124] (reproduced, with permission, from [127]).

The metabolism of radiolabelled 20E has been more extensively studied [126,127]. These studies involved 20E radiolabelled in the nucleus, so as to be able to follow side-chain cleavage. It was found that the fate of ingested 20E was initially simple, with most of the 20E remaining unaltered as it passed through the esophagus, stomach and small intestine, with only a small proportion crossing into the blood and being captured by the liver (Figure 7).

After ca. 30 min, 20E starts to reach the large intestine, where it begins to be significantly metabolized primarily to 14d20E. Further, the 20E and 14d20E start to undergo complex metabolism including side-chain cleavage. Absorbed 20E and its metabolites are taken by up the liver, enter the bile duct and are brought back to the intestine. Entero-hepatic cycling then occurs, during which 20E and the metabolites leave the gut, enter the blood, are taken up by the liver and returned through the bile duct to the intestine. This result is fully consistent with the observations of Wu et al. [124] of a sustained high radioactivity in bile for up to 12 h (Table 3). As a consequence, the duration of 20E presence in the plasma is prolonged at a low, but physiologically significant, concentration (in the μM range—depending on the dose administered), and the extent and complexity of the metabolism of 20E increase markedly, such that the excreted feces contain unaltered 20E and a wide range of metabolites, produced by permutations of several biochemical reactions.

**Figure 8:**
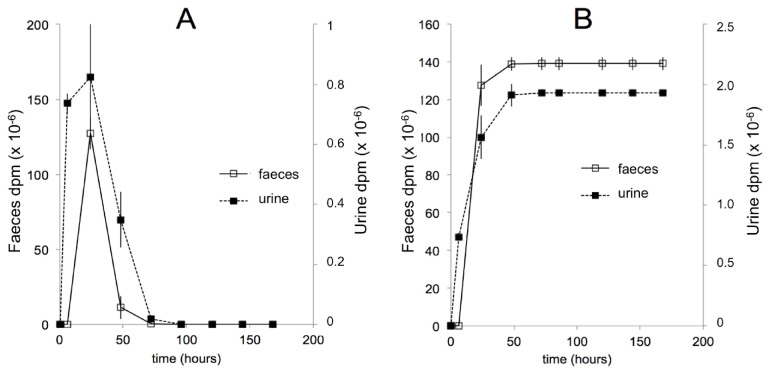
Comparison of the temporal (**A**) and cumulative (**B**) urinary and fecal elimination of radioactivity after the oral application of [5,7,9-^3^H]20E to 6–7 week-old male Wistar rats. After oral application of [^3^H]20E, elimination of radioactively labelled 20E and its metabolites is completed within 48 h. The amount of radioactivity recovered from the feces is by far the major route of excretion, since the amount in the urine corresponds to only 1.40% of the total radioactivity recovered. (Reproduced, with permission, from [127]).

The bioavailabilities of 20E (ca. 1%) and Post (ca. 19%) have been determined [127,128]. As in mice, the initial fate of ingested 20E is uncomplicated, remaining almost completely associated with the gut in unchanged form, until it reaches the large intestine, where microbial metabolism and metabolism associated with the enterohepatic cycle come into play to rapidly increase the extent and diversity of metabolites. Evidence has been provided that glucuronide conjugates of 20E are present in the bile fluid, but these are hydrolysed on reaching the large intestine. The major metabolites have been conclusively identified as 14d20E, Post, 14dPost, 3-epi-Post, 16αOHPost, and 21OHPost [128], but other permutations of these biochemical reactions almost certainly exist. Additionally, other metabolites where the 6-one-7-ene chromatophore has been modified, so that the products are no longer UV-absorbing, are likely to be present amongst the complex mixture of metabolites present in the large intestine and excreted in the feces. The elimination of ingested or injected 20E is predominantly fecal (Figure 8A,B). The microbial origin of the 14d20E metabolite is demonstrated by the absence of this metabolite in axenic rats fed 20E [129] and by its formation from 20E in incubations of intestinal contents under anaerobic conditions [127]. That 14-dehydroxylation is associated with the microbiote of the large intestine is in accord with previous findings that dehydroxylations of steroids and bile acids are only bacterial [130,131]. The locations of the other metabolic reactions are not currently known with certainty, but it is expected that they will be associated with specific enzymes of vertebrate steroid catabolism.

## 11. Pharmacokinetics and Metabolism in Humans

### 11.1. Early Studies

Although ecdysteroids are not biosynthesized in mammals, sensitive methods can detect them in mammalian body fluids (plasma, urine). Thus, ecdysteroid-specific radioimmunoassay (RIA) was used to detect immunoreactive ecdysteroids in the serum of seven mammals (dog, rhesus monkey, sheep, cow, rabbit, mouse and rat) and humans in nM concentrations [132], which originate from the food. Ingested ecdysteroids (20E or E) reach a plasma titre maximum after ca. 30 min in humans and sheep and are rapidly cleared from the blood and urine, but the proportions of the applied dose found in these are very low, accounting only for a few percent. The effective half-time of elimination from the blood in humans was found to be 4 h for E and 9 h for 20E, and 3.1 h for E and 3.3 h for 20E for elimination from the urine [132].

Pharmacokinetic studies in humans are more restricted owing to the limitations on the use of radiolabelled molecules. Consequently, only a few 20E pharmacokinetic studies have been performed. In humans, Simon and Koolman [132] showed that ingestion of ca. 15 mg 20E induced a urinary peak level of approximately 0.5 μM. Significant immunoreactivity was detected during the first 8 h following intake, but weak urinary levels were still observed until 24 h after ingestion [133]. After a single oral intake of 20 mg 20E, a more detailed study by RIA and liquid chromatography-mass spectrometry (LC-MS) showed the existence in urine of a peak of detectable ecdysteroids after 3–4 h and a total quantity of 1–2 mg—the exact nature of the compounds was not fully determined, although one could be tentatively identified as 14d20E. In another study [134], the ingestion of 434 mg 20E resulted in the urinary excretion of a total of 5 mg of 20E, corresponding to approximately 1% of the ingested amount. This study also indicated that urinary excretion seems somewhat more significant in humans than in rodents [135], even though the fecal content was not quantified and small amounts were recovered from urine as compared to the administered dose.

### 11.2. Recent Studies

**Figure 9:**
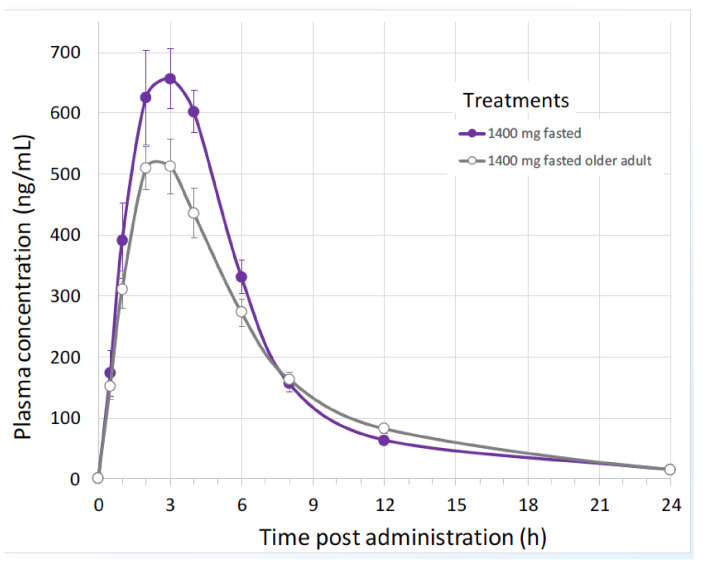
Pharmacokinetics of 20E in young and elderly humans given a single dose of 1400 mg. 20E was quantified by HPLC-MS [136,137].

Biophytis has recently undertaken the first in-human, randomized, double-blind, single-centre study to determine the safety of 20E and its major metabolites in healthy young (18–55 years) and elderly (>65 years) adult subjects [136,137]. The study comprised two parts: a single ascending dose component (SAD; 16 subjects) and a multiple ascending dose component (MAD; 30 subjects). 20E showed a good safety and pharmacokinetic profile, which allowed the doses for a Phase 2 Sarcopenia clinical trial to be defined. Pharmacokinetics in subjects treated with increasing amounts of orally delivered 20E (Table 4) showed that the level found in the plasma is dose-related and also apparently saturable. The kinetics of plasma 20E was similar between young and older subjects (Figure 9).

About 2% of the administered 20E was excreted in the urine within 12 h of ingestion of a single dose, and half of this was excreted within 4 h. 20E and its metabolites were identified and quantified in the plasma of individual patients who had received oral 20E. These metabolites corresponded to the major ones previously found in rodents (14dPost, 14d20E and Post).

## 12. The Major Routes of 20E Metabolism in Rodents and Humans

**Figure 10:**
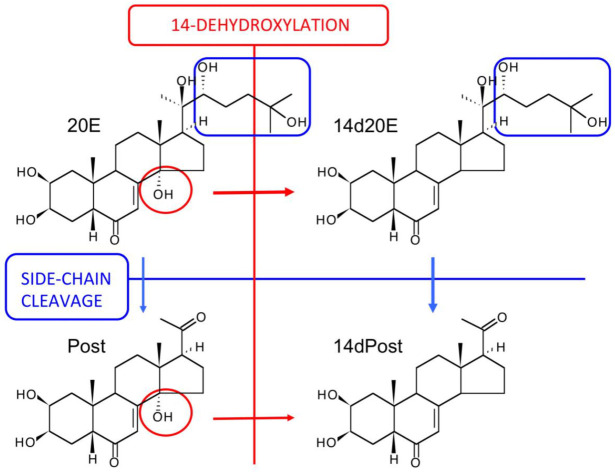
The principal routes of metabolism of 20E in rodents and humans (modified from Kumpun et al., 2011). The circles and arrows in red highlight the changes associated with 14-dehydroxylation, while the boxes and arrows in blue highlight the changes associated with side-chain cleavage.

Figure 10 summarizes the metabolic reactions currently known to occur for 20E in mammals to produce a complex array of metabolites arising from combinations of the metabolic possibilities, although the flux through the network of combinations will depend on the exposure to and the activity of the different enzymes depending on the mammalian species. On ingestion, bioavailability is very low, as is initial metabolism, and it is only on reaching the large intestine that 14-dehydroxylation occurs, but this is a reaction performed by the gut flora. Only ecdysteroids with a 20,22-diol (such as 20E) undergo side-chain cleavage, as demonstrated by the absence of this reaction with oral or injected E. Side-chain cleavage and 14-dehydroxylation significantly enhance bioavailability (see below) and an enterohepatic cycle maintains ecdysteroid plasma levels and enables more extensive metabolism (e.g., hydroxylations, reductions, epimerizations—the two latter already observed with ecdysone [125]).

## 13. Large-Scale Production of Pure 20E for Drug Development

### General Considerations

Various theoretical possibilities for ensuring the supply of 20E in adequate amounts and at reasonable cost have to be considered:

1. Total chemical synthesis
2. Isolation from the current plant source, e.g., *Cyanotis* sp. following cultivation and collection in China (Yunnan province)
3. Cultivation of another species already identified as a good accumulator with a simple ecdysteroid profile (*Rhaponticum*, *Serratula* or *Pfaffia* sp.)
4. Use plant cell or hairy-root cultures of an ecdysteroid-producing plant
5. Generate recombinant yeasts using insect and/or plant enzymes of ecdysteroid biosynthesis.

Of these, possibilities 1 and 4 can be discounted; 1 because of the complexity (≥18 steps) and poor overall yield (ca. 1%) of the process, and the rate-limiting access to a suitable starting material, and 4 probably because of the high cost of the media and low yields normally attained for plant secondary compounds, even when using elicitors [138]. Various researchers have started to explore a range of culture methods in vitro (e.g., micropropagation, callus cultures, plant cell culture, hairy-root cultures, transformed yeast fermentation) in the hope of obtaining more amenable ecdysteroid-producing systems. To date, only hairy roots of *Ajuga reptans* var. *atropurpurea* (Fujimoto et al. [139]) consistently and reliably produce ecdysteroids, but the culture is too expensive to provide a commercial source of 20E. Additionally, hairy roots of some other ecdysteroid-producing species do not contain ecdysteroids, so this cannot be viewed as a general approach.

Possibility 5 will for sure face a number of difficulties, but, once established, it would provide relatively easy scale-up. The use of modified yeasts to produce bioactive molecules is rapidly developing [140] and proved efficient even for multiple-step biosyntheses (e.g., [141]). Transforming yeast to biosynthesize ecdysteroids, as has been previously performed for certain vertebrate steroids [142,143], is an attractive prospect, but is currently confounded by our incomplete knowledge of the ecdysteroid biosynthetic pathways in either invertebrates or plants and the probable high number of genes involved in the complete pathway(s) [3].

Possibility 2 may make the supply dependent on a single country and on the difficulty to secure access to the material in case of competition for a limited supply. Possibility 3 might displace the problem to another country and requires the development of a large-scale culture.

At the moment, the molecule is extracted from suitable plant materials. It is perhaps worth reminding ourselves of the criteria that an ideal plant source should fulfil:

- The plant should accumulate a high amount of 20E
- The plant should have a simple ecdysteroid profile (ideally just 20E)
- The plant should be easy and rapid to grow in accessible areas of the world
- The plant matrix should be amenable to the ready purification of ecdysteroids
- The purification and isolation of 20E should not involve expensive chromatographic methods
- The plant should not be susceptible to pests and diseases
- The species should not be rare or protected
- Culture, harvesting and processing costs should be low; initial processing should take place close to the culture site

Clearly, no plant species will fulfil all these criteria, but the closer it comes, the more culturally and comercially viable the challenge of isolating adequate amonts of 20E will become. Major screening of plants for the presence of ecdysteroids began by using very time-consuming, semi-quantitative whole insect bioassays (*Chilo* dipping test; *Musca* assay; *Sarcophaga* assay), moved on to the combined use of ecdysteroid-specific RIAs and a microplate assay based on an ecdysteroid-responsive cell line, and most recently progressed to analysis by HPLC/DAD/MS-MS, which allows the simultaneous identification and quantification of common phytoecdysteroids and dereplication to detect the presence of previously unknown ecdysteroids [144].

Most ecdysteroid-accumulating species contain between 0.01 and 0.1% of the dry weight as ecdysteroids, whereas the few identified high-accumulators contain 1% and above. The roots of *Cyanotis* spp. can contain up to 5% of the dry weight as ecdysteroids, largely as 20E [145]. Mature stems of *Diploclisia glaucescens* were found to contain 3.2% of their dry weight as 20E [146], but the collection of the stems of this South Asian climber precludes this as a feasible source of large amounts of 20E.

## 14. Plants Species Presently Used to Produce Purified 20E

The relationship between the presence of phytoecdysteroids and plant taxonomy is complex, but high accumulation is associated with certain species in the genera *Achyranthes* (Amaranthacea), *Cyanotis* (Commelinaceae), *Pfaffia* (Amaranthaceae), *Rhaponticum* (syn. *Leuzea*/*Stemmacantha*; Asteraceae), and *Serratula* (Asteraceae), which can be considered as good sources of 20E.

***Achyranthes*** (*A. aspera*, *A. bidentata*, *A. fauriei*, *A. japonica*): Perennial or HHA; warm temperate and sub-tropical plants; native to South-east Asia and/or Africa; all plant parts contain ecdysteroids with seeds containing the highest level (0.25%) in *A. aspera* and roots (1.74%) being the best source in *A. bidentata*;
***Cyanotis*** (*C. arachnoidea*, *C. vaga*): Perennial; tropical and sub-tropical plants; growing in tropical Africa, the Indian subcontinent and southern China; roots accumulate very high levels of 20E (up to 5.5% of the dw; Wang et al. [145]); simple ecdysteroid profile—major source for commercial 20E cultivated on a large scale in China;
***Pfaffia*** (*P. glomerata*, *P. iresinoides*): Perennial; tropical plants; native to South America; *P. glomerata* contains ecdysteroids throughout the plant; roots have a very simple ecdysteroid profile, consisting solely of 20E (0.9% of the dw);
***Rhaponticum*** (*R. carthamoides*): Perennial; sub-alpine plant; native to the Altai and Sayan Mountains in Central Asia; roots, flowers and seeds accumulate high levels of 20E (1–2% of dw); complex ecdysteroid profile, but >80% as 20E—used to prepare ECDYSTEN pills containing 5 mg of 20E (OPIH, Uzbekistan) [147];
***Serratula*** (*S. centauroides*, *S. coronata*, *S. tinctoria*, *S. wolfii*): Perennial; temperate plants; native to western and central Europe; *S. tinctoria* accumulates up to 2% of dw as ecdysteroids; the major ecdysteroids are 20E 3-Acetate, 20E and PolB; *S. coronata* is used to prepare SERPISTEN, a 8:1 mixture of 20E and 25*S*-inokosterone [148].

We should mention that only 2% (ca. 6000) of terrestrial plant species have been assessed for the presence of phytoecdysteroids and far fewer than this have undergone extensive phytochemical analysis to identify the ecdysteroid analogues present [144]. Thus, there are certainly many other as yet unidentified species which are high accumlators of 20E, but it has to be borne in mind that the species already tested represent the most readily available species and accessing samples of new untested, but validated, species would require the investment of a large amount of time and effort.

Owing to the wide taxonomic diversity of plant species known to accumulate phytoecdysteroids, it has been proposed that most, if not all, plants have the genetic capacity to produce ecdysteroids, but in the majority (the non-producers) the expression of the biosynthetic pathway is down-regulated [2]. Additionally, it has been shown in several ecdysteroid-accumulating species that ecdysteroid levels are additionally influenced by environmental factors (temperature, nutritional factors, invertebrate predation, etc.) [149,150]. Thus, to maximize 20E content in a chosen species, it is not only necessary to select a high producing genetic line (cultivar), but also to optimize the growth conditions and to consider treating the plants with appropriate elicitors (e.g., methyl jasmonate to mimic insect or fungal attack) at suitable times in the development.

## 15. Purification Process

The major requirements in the processing of plant material to obtain a drug product in adequate amounts for therapeutic use are simplicity, efficiency of extraction and purification, reproducibility and cost-effectiveness. As explained above, the choice of plant material is key, since not only should it contain a large amount of the target molecule, but the plant matrix should be readily extractable and not contain components (e.g., large amounts of polysaccharides) which make processing difficult. Given that a suitable plant source will contain typically 1–2% of the dry weight as 20E, and that a pharmaceutical/medicinal dose of 20E is likely to be in the 100 mg–1 g/day range, it is necessary to be able to process tonnes of plant material. Clearly, it would be highly advantageous if the harvested plant material can be cleaned, dried, broken up (to reduce volume and increase the surface area) and subjected to initial extraction as close to the site of harvesting as possible, as the mass of the initial extract is probably only 1–2% of the fresh weight of the plant material and, therefore, much more readily and cost-effectively transportable.

The extraction and purification have to be optimized with regard to the physico-chemical properties of the target molecule (generally already known or readily determinable) and the nature of the plant matrix (generally only vaguely understood). Owing to the large number of hydroxyl groups in 20E, it is highly soluble in alcohols, so the dried, powdered plant material will usually be extracted with methanol (preferred as it is cheaper) or ethanol (if the product is to be BIO/Organic), with or without prior extraction with a non-polar solvent, such as petroleum ether, to de-fat the plant material. Extraction may occur with heating, stirring or maceration for a defined time, all of which need to be optimized. Other methods, such as super critical fluid extraction or bi-phasic extraction, may be used, but these are generally more expensive and would only be cost-effective if the target molecule is potent (low daily dose required) and high-value, which is not the case for 20E.

**Figure 11:**
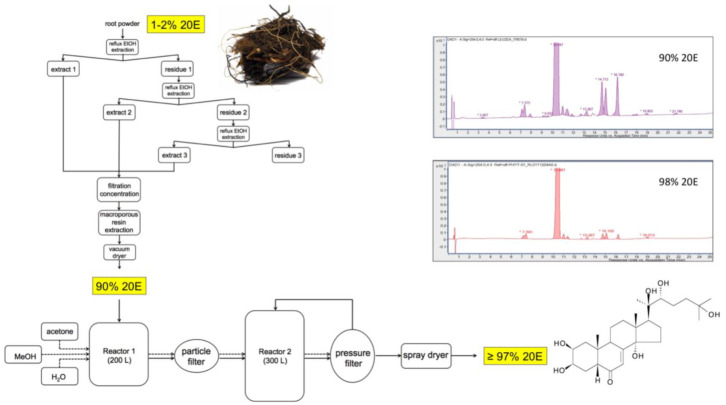
A representative flow-diagram for the large-scale extraction and purification of 20E from roots of *Cyanotis* sp.

Figure 11 provides a flow-diagram of a representative processing method for *Cyanotis* sp. roots, where the dried plant material is refluxed thrice with ethanol and the pooled extracts are filtered before being passed through a macroporous resin to absorb plant compounds and vacuum-dried to yield a powder containing 90% 20E. This preparation can then be recrystallized twice to bring the purity of the 20E to >97%.

A comparison of the reversed-phase (RP)-HPLC profiles of the 90% and 98% 20E preparations is shown in the inset to Figure 11. Most of the minor peaks in the chromatograms correspond to other ecdysteroids, which are very difficult to separate fully from 20E by crystallization. This underlines the need to start with plant material which contains essentially only 20E. Whenever the plant contains a high proportion of a very close molecule (e.g., inokosterone or polypodine B), it would be very difficult to achieve the required purity.

Owing to the need for optimization at each stage of the extraction and purification, the process is developed in stages going from small-scale (100 g—a few kg dry plant material) in the laboratory through increasing medium-scale stages (e.g., 10–500 kg) before being applied at the industrial scale (>1 tonne), so that difficulties can be identified and resolved early on and any problems of scale-up can be dealt with. A thorough cost analysis needs to be performed throughout the scale-up procedure to ensure that the target molecule can be brought to market at a viable cost.

## 16. Quality Control and Stability

Using 20E as the active ingredient of a pharmaceutical grade drug candidate requires the complete control of the raw material (roots, aerial parts or whole plant harvested from the field) following the GAP (Good Agricultural Practices; http://www.cfsan.fda.gov/~dms/prodguid.html, accessed on 1 April 2021) or the guidelines on the quality of herbal medicinal products/traditional medicinal products. This involves the description of the active substance preparation including geographical origin, cultivation, harvesting, and postharvest treatments (possible pesticides and fumigants used and/or formation of genotoxic impurities, solvents, presence of aflatoxins, traces of heavy metals and possible radioactive contamination).

The pharmaceutical grade compound (purity ≥ 97%) must be fully characterized, and all its impurities ≥ 0.1% must be identified, with none of them being above 0.5%, otherwise it would require specific toxicological studies. In the present case, given the process used and the fact that ecdysteroid-containing plants contain a complex cocktail of these molecules (e.g., [26,151]), most remaining impurities correspond to other phytoecdysteroids, the nature of which will depend on the plant used.

Compound stability studies also have to be performed, which does not raise problems in this case, as ecdysteroids are very stable molecules when stored in dry state in the dark at room temperature.

## 17. Regulatory Preclinical Studies

### Preclinal Regulatory Requirements

Nutritional studies can be allowed with limited prerequisite regulatory elements, provided that 20E comes from an edible plant (e.g., spinach, quinoa or *Leuzea*—a plant on the Belgian list) and the amounts administered are not too high as compared with those present in a diet comprising the above plants (e.g., 50 g quinoa contains 20–30 mg 20E). Such studies were performed in relation to anabolic or anti-obesity effects on healthy subjects.

On the other hand, clinical studies aimed at treating human diseases with possibly higher doses and for extended periods require a lot of information about the molecule administered and its long-term effects on model animals, typically one rodent and one non-rodent species (Table 5).

Safety pharmacology studies must thus be conducted to investigate acute effects on the central nervous system, respiratory function, cardiovascular system including hERG potassium channel on blood pressure, heart rate, core body temperature and electro-cardiogram.

Long-term toxicity studies also have to be performed in one rodent and one non-rodent species, the duration of which must correspond to the expected duration of administration during the clinical trial.

## 18. Regulatory Studies with 20E Metabolites

20E undergoes an extensive metabolism after administration, in part at least performed by the gut microbiote, and probably also in the liver, which is known to metabolize endogenous steroid hormones. Whenever metabolites would circulate at significant levels (area under the curve (AUC) ≥ 10% that of 20E), regulatory studies would be required to assess their lack of toxicity. In addition, it is relevant to determine their biological activity, i.e., whether they have a similar/lower/higher activity to that of the parent compound, possess a different (specific?) activity, or if they just represent inactivation products.

### 18.1. Some Drugability Calculations

As indicated above, the bioavailability of 20E is low, while that of its metabolites may be significantly higher. In a first step to relate the bioavailability of ecdysteroids to their physicochemical properties and structures, we have calculated the ADMET Traffic Light Scores for ecdysteroids according to Lobell et al. [152] based on values for MW, LogP, the polar surface area (PSA), and the number of rotatable bonds. However, rather than using solubility (which has to be experimentally determined), the number of N + O atoms (as a measure of H-acceptors) and the number of NH + OH groups (as a measure of H-donor groups) were used as in the original method of Lipinski et al. [153]. The procedure generates a score (explained in the legend of Table 6) between 0 and 10 for each compound; the lower the value, the more suitable the physicochemical properties are for an orally administered drug.

Each parameter (MW, LogP, PSA, Rotatable Bonds, H-acceptors and H-donors) was scored as described in the legend to Table 6. The allocated score is represented by the background colour in the above table: 0 = green, 1 = orange and 2 = red. The scores are added to give the Traffic Light Score (0–10), where 0–2 is deemed green (good drugability), 3–6 orange (moderate drugability) and 7–10 red (poor drugability).

Table 6 indicates that, once formed, C_21_-metabolites should be readily absorbed from the gastrointestinal tract, since they all have a TL Score of zero, whereas 20E and most of its C_27_-metabolites should be absorbed far less readily (with scores of 4 or 5). The only exception to this is 14d20E (which is the major initial intestinal metabolite of ingested 20E), with a TL Score of 1, owing to its lower PSA and lower number of H-bond donor groups after the removal of the 14α-hydroxyl group. This is in accord with the experimental observation that this metabolite is found in the urine of rats and mice after the ingestion of 20E, implying that at least a portion of the 14d20E formed in the large intestine is absorbed into the blood to reach the kidneys [127].

### 18.2. SAR Studies

Only a few structure-activities studies are already available for some natural phytoecdysteroids (e.g., [35,38,47]), but almost no data are available for the above-mentioned metabolites, except for Post and for rubrosterone, a potential C_19_ metabolite not yet observed in metabolic studies. Both Post and rubrosterone seem to retain some anabolic activity [154,155]. Thus, data are still lacking for 20E metabolites.

For those major human metabolites, specific toxicological studies may be required, unless they would be formed in similar amounts in an animal model. Such studies may include off-target binding studies as previously performed with 20E [113]. In addition, it appears relevant to assess their biological activity at least in vitro in order to understand whether they participate to the overall effects observed in vivo.

## 19. Clinical Studies

Early experimental studies and clinical trials on humans have indicated that 20E or plant extracts containing significant amounts of ecdysteroids [104]:

- Prevent sleep disorders [156]
- Improve nerve functioning in the CNS [157]
- Improve sexual function [158]
- Improve hepatic function in hepatitis [159]
- Improve learning and memory [160]
- Improve cardio-vascular function [161]

A major aim of early human studies was to improve physical performances of healthy young people, and they showed a significant improvement in muscle mass and/or endurance after a few days/weeks. Such experiments paved the way, directly or indirectly, for use as a “doping substance” by sportsmen or bodybuilders based on the consumption of uncontrolled dietary supplements [162]. The recent confirmation of these anabolic results [163] led the authors to propose the inscription of 20E on the list of doping prohibited substances [164].

We have chosen to concentrate here on trials aimed at improving human health from a medicinal perspective using pure 20E, as concentrated plant extracts might contain other bioactive substances, e.g., flavonoids. Those selected trials are summarized in Table 7.

## 20. Neuro-Muscular Diseases

### Sarcopenia

Sarcopenia is an age-related skeletal muscle disorder characterized by a progressive loss of muscle mass and strength that leads to reduced mobility. Sarcopenia also has a neuronal component linked to the progressive death of motoneurones [178,179]. 20-Hydroxyecdysone has previously shown beneficial effects in several disease animal models including aging/sarcopenia. It has been purified to pharmaceutical grade (≥97% purity = BIO101) for use as a drug candidate and proved safe in rat and dog models upon chronic treatment. For Single Ascending Dose (SAD) study, BIO101 was administered orally to 24 subjects from two age groups: young adults (18 ≤ age ≤ 55 years) at escalating doses (100 to 1400 mg), and older adults (65 ≤ age ≤ 85 years) at 1400 mg. For Multiple Ascending Dose (MAD) study, three doses of BIO101 (350 mg once a day; 350 mg twice a day and 450 mg twice a day) were administered to older adults over 14 days. The primary objective was to evaluate the safety and pharmacokinetics of BIO101 and to identify its main metabolites. The effects of BIO101 were also investigated by measuring selected biomarkers during MAD. BIO101 showed a good safety profile with no serious adverse events during SAD and MAD. All adverse events were of mild or moderate intensity. The bioavailability of BIO101 was rather low, and plasma levels increased less that dose-proportionally. Plasma BIO101 half-life ranged between 2.4 h and 4.9 h, and mean renal clearance ranged between 4.05 and 5.05 L/h. Several metabolites were identified and measured in plasma. Elimination used mainly the fecal route. After 14 days of administration, serum biomarkers of muscular catabolism (myoglobin, CK-MB) were reduced in subjects administered the highest dose. This Phase 1 study confirmed the safety of BIO101 and allowed us to define the most appropriate oral doses for the ongoing interventional Phase 2 clinical trial (ClinicalTrials #NCT03452488) [180].

SARA-INT is a Phase 2 interventional study performed in Europe and the USA aimed to evaluate the clinical benefits, safety and tolerability of the investigational drug BIO101 administered orally for a six-month (26 weeks) duration to older patients, community dwelling men and women aged ≥65 years, suffering from age-related sarcopenia (including sarcopenic obesity), and at risk of mobility disability. The double-blind, placebo controlled clinical trial will collect and analyze data on physical performance and body composition and will specifically focus on the change of one functional measurement, the gait speed measured during the 400 metre walking test plus the change of a highly standardized patient reported outcome, the physical function domain PF-10 at the SF-36 auto-evaluation questionnaire, in order to estimate the efficacy of BIO101 administered over 26 weeks in preventing mobility disability in the target population [173].

## 21. Cardio-Metabolic Diseases

### 21.1. Pre-Diabetes

Prediabetes is the term used for people whose glucose levels do not meet the criteria for diabetes but are too high to be considered normal. This is defined by the presence of blood glucose between 100 and 125 mg/dL, values per glucose tolerance curve of 140–199 mg/dL and/or HbA1c 5.7–6.4%. Prediabetes should not be considered as a clinical entity in itself, but as a risk factor for diabetes and cardiovascular disease. Prediabetes is associated with obesity (especially abdominal or visceral obesity), dyslipidemia with elevated triglycerides and/or low HDL cholesterol, and hypertension. Subjects with a diagnosis of prediabetes are included according to the criteria of the American Diabetes Association in its version 2019, between 30 and 60 years old and residents of the city of Guadalajara, Jalisco, Mexico who come to clinical nutrition consultation in the University Hospital Fray Antonio Alcalde from the city of Guadalajara, Jalisco, Mexico. The study design is a randomized clinical trial with a control group in two groups: an intervention group with 20E 300 mg every 24 h for 12 weeks and an approved placebo control group (magnesium stearate) at 300 mg every 24 h for 12 weeks [175].

### 21.2. Obesity

20E prevents obesity and osteoporosis following ovariectomy in rats. Whether it also protects joint cartilage was compared with the effects of estradiol (E2). Furthermore, the effects of 20E in 20 slightly overweight male and female persons were also determined. In ovariectomized rats, 20E reduced the amount of abdominal, bone marrow and joint fat (*p* < 0.05), and this resulted in better trabecular and joint cartilage architecture. In the open clinical trial with slightly overweight persons, a daily intake of 200 mg 20E reduced waist circumference by 2.7 cm, serum cholesterol by 10.2%, LDL by 13.9% and triglycerides by 31.1%, whereas HDL increased by 10.8%. It is concluded that 20E has similar osteo- and chondro-protective effects as E2 and prevents fat accumulation in the abdomen, bone marrow and joints. Hence, 20E may prevent metabolic syndrome and accompanying osteoporosis and osteoarthritis [171,172].

### 21.3. Menopause

Many postmenopausal women, but increasingly also young females and males, develop obesity. Today, two types of obesity are differentiated: the gynoid “pear” type with fat large gluteal and thigh fat and the android “apple” type with large visceral fat depots. Metabolic syndrome develops primarily in obese people with large visceral fat depots. Adipocytes of the visceral type secrete proinflammatory cytokines which, in the apple-type obese, set the whole body in an inflammatory condition with high oxidative stress with harmful effects in many organs including the arteries, which results in hypertension. Insulin receptors are desensitized, leading to the development of type-2 diabetes. Obese persons also suffer often from hyperlipidemia, which may lead to arteriosclerosis and heart attacks. Metabolic syndrome is not only dangerous for the cardiovascular system, but also for bones, joints, and musculature because there are accumulating fat cells in bone marrow, fat pads in joints, and adipocytes in muscles also secrete proinflammatory cytokines which inhibit the formation of bone osteoblasts, cartilage chondroblasts, and muscle myoblasts. In bone, cytokines even stimulate the formation of bone-resorbing osteoclasts. These effects of adipocytes result in epiphenomena of metabolic syndrome namely in osteoporosis, arthrosis, and sarcopenia. 20E has proven inhibitory effects on the formation of adipocytes. Therefore, 20E is able to reduce the amount of body fat and thereby simultaneously inhibit the reduction in muscle, bone and joint cartilage [172].

### 21.4. Metabolic Syndrome

The aim of the study was to analyze the interactions between body fat, especially visceral fat depots, on parameters such as high-sensitivity C-reactive protein (hsCRP) and the lipid profile. The effects of 20E on the body parameters fat percentage, muscle mass, body weight and abdominal circumference as well as on the serum parameters cholesterol, triglycerides, LDL, HDL and the hsCRP of obese people with metabolic syndrome were retrospectively investigated. The control group included data from a patient population with metabolic syndrome who did not take 20E. In addition, the vitamin D metabolism of obese people was analyzed. From this retrospective study, it is clear that the abdominal circumference, as an indicator of visceral obesity, correlates positively with TG and hsCRP. Patients with an abdominal circumference of ≥100 cm have vitamin D deficiency, whereby the deficiency correlates positively with the size of the abdominal girth. By taking 20E, a continuous and significant loss of body mass, fat percentage, and waist diameter can be achieved while preserving muscle mass in patients with metabolic syndrome compared to the control group. 20E has no significant effect on serum lipids. In contrast, the hsCRP level undergoes a marked reduction. In summary, 20E exerts a positive influence on body parameters, in particular the visceral fat depot and on the hsCRP level. Influence on the lipid profile of patients with metabolic syndrome requires further investigation. Patients with a high visceral fat depot are also exposed to a risk of vitamin D deficiency or undersupply [174].

## 22. Respiratory Diseases

### 22.1. Respiratory Failure in COVID-19 Patients

**Figure 12:**
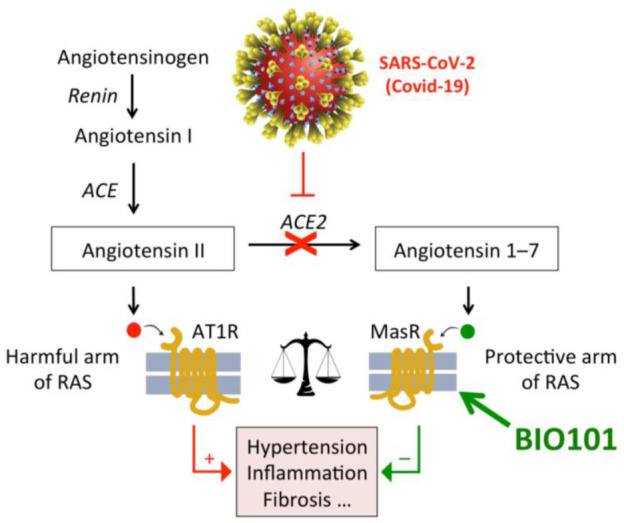
SARS-CoV-2 infection will result in a strong impairment of the activity of angiotensin converting enzyme 2 (ACE2), hence a lack of angiotensin-(1-7) production and a disequilibrium between the harmful and protective arms of the renin–angiotensin system (RAS). Treatment with BIO101 (20E) is expected to activate Mas receptor and the protective arm of RAS, thus preventing inflammation and lung damage.

The clinical picture of COVID-19 including acute respiratory distress syndrome (ARDS), related to interstitial pulmonary fibrotic inflammation, cardiomyopathy and shock, strongly suggests that the renin–angiotensin system (RAS) is dysfunctional owing to interaction between angiotensin converting enzyme 2 (ACE2) and SARS-CoV2 spike protein. It is proposed that RAS balance could be restored in COVID-19 patients through Mas1 activation downstream of ACE2 activity, with 20E (BIO101), a non-peptidic Mas receptor (Mas1) activator developed by Biophytis (Figure 12). Indeed, Mas1 activation by 20-hydroxyecdysone harbours anti-inflammatory, anti-thrombotic, and anti-fibrotic properties. BIO101, a 97% pharmaceutical grade 20E, could then offer a new therapeutic option by improving the respiratory function and ultimately promoting survival in COVID-19 patients that develop severe forms of this devastating disease. Therefore, the objective of this COVA study is to evaluate the safety and efficacy of BIO101 in COVID-19 patients with severe pneumonia [180].

### 22.2. COVID-19

The COVA clinical study is a global multicentric, double-blind, placebo-controlled, group sequential and adaptive two parts phase 2–3 study targeting patients with SARS-CoV-2 pneumonia. Part 1 is a Phase 2 exploratory Proof of Concept study to provide preliminary data on the activity, safety and tolerability of BIO101 in the target population. Part 2 is a Phase 3 pivotal randomized study to provide further evidence of the safety and efficacy of BIO101 after 28 days of double-blind dosing. BIO101 is the investigational new drug that activates the Mas1 through the protective arm of the renin–angiotensin system (RAS) [176,181].

## 23. Other Diseases

### 23.1. Hepatitis

The inclusion of Ecdysten into a complex therapy scheme for patients with chronic viral hepatitis B (5-mg tablets twice per day over a period of 30 days) substantially improved the clinical and biochemical indices of the functional state of the liver, positively influenced the humoral, cell immunity and the resistance factors, and normalized the course of autoimmune processes accompanying the liver pathology [159].

### 23.2. Chronic Glomerulonephritis

The administration of Ecdysten (3 tablets/day (=15 mg 20E/day) for 10 days) to patients (18 vs. 17 controls) with chronic glomerulonephritis (inflammation of the glomeruli of the kidney) improved the morphometric indices of the microcirculation of the bulboconjunctiva in the majority of patients examined. The frequency of vascular, intravascular and perivascular changes in the bulbar conjunctiva decreased. The frequency of twisting, irregularity, aneurism, and reticulation of the vessels and the phenomena of stagnation of the venous networks and of zones of degeneration fell substantially. In addition, a distinct tendency to a normalization of the diameter of the arterioles, capillaries, and venules appeared. The most pronounced effect was observed in patients with the nephrotic form of the disease [166].

### 23.3. Celiac Disease

This autoimmune disorder is due to gluten intolerance and primarily affects the small intestine, causing chronic diarrhea, impaired absorption and loss of appetite. It is expected that reduced intestinal absorption is caused by energy metabolism impairment in intestinal cells, and that it results in overall negative effects on general metabolism [177]. The treatment with Ecdysten for 2 weeks resulted in a tendency to improve energy metabolism as assessed by lowered lactic acid plasma concentrations.

### 23.4. Sexual Dysfunction

Ecdysten has been shown to improve sexual function in men suffering from low sperm count, poor sperm motility, erectile dysfunction or low libido when recovering from heart attack [158,165].

### 23.5. Parasitoses (Giardiasis, Hymenolepiasis, Lambliasis)

This area has been documented by several trials using Ecdysten [167,168,169]. We expect that the mechanism involved here is totally different, as it is most probably a direct effect of 20E on the parasites themselves.

## 24. Conclusions and Prospects

20E shows many beneficial pharmaceutical effects in mammals and is non-toxic. To date, the only ecdysteroid which has been investigated to any extent is 20E, for the simple reason that it is the only analogue which is readily commercially available at a reasonable cost. Where it has been investigated, the effects are brought about by low μM concentrations of 20E, but to achieve maintained concentrations of this level in the plasma it is necessary that 20E should be supplied orally in large amounts (100–1000 mg) at least twice a day, because the bioavailability of 20E is poor and the half-life in the plasma is short. In the future, it will be necessary to seek ways to overcome the poor bioavailability by alternative application routes (e.g., nasal) and improve formulation by identifying biologically active analogues with greater inherent bioavailability. It is fortunate that a wide range of ecdysteroid structural analogues are already known (Ecdybase [4]) and samples of many of these are available by isolation from plants or semi-synthesis, at least in the amounts required for method development and structure-activity relationship studies. Thus, it is to be expected that one of the areas of focus in the near future will be the development of more amenable and specific bioassays for each of the major effects of ecdysteroids in mammals, leading to a clearer understanding of the SARs for activity, potency and uptake from the gut.

A further driving force for such studies will be the growing need for an increased supply of 20E, since extraction from natural plant sources is a viable approach for kg amounts of pure substance (as required for thorough clinical trials), but it is difficult to envisage how this could produce multi-tonne amounts of 20E (as could be required to treat sarcopenia or metabolic syndrome on a world-wide basis). This will provide a spur to seek ways of elevating 20E accumulation in ecdysteroid-accumulating plants above the 1–2% of the dry weight typical of the sources currently used, or to develop much more efficient and greener chemical syntheses of 20E or its analogues or molecular approaches transforming yeast and developing high-yielding lines for fermentation, and to seek more effective analogues and better ways of delivery. Fundamental to both the improved yield in plants and the yeast fermentation is a thorough understanding of phytoecdysteroid biosynthesis, not only the biochemistry and molecular biology of the individual steps, but also the integration and regulation of these steps to form the complete pathway(s) and to understand the flux and dynamics through the pathway to maximize 20E formation, all of which are currently only sketchy.

In the past, the most extensively studied effect of 20E in mammals was the anabolic effect, which was exploited initially rather empirically to improve the stamina and performance of sportsmen and -women, but is now starting to gain a mechanistic understanding. The regulatory agencies have recently started to analyze those uses and those will possibly become prohibited in the near future. 20E is presently included in the 2020 Monitoring Program of the World Antidoping Agency (WADA [182]). They do not represent the most promising uses for this molecule, and the recent clinical trials aim to treat different human diseases with a rationale based on 20E mechanism of action.

Indeed, the protective arm of the RAS system has a wide number of target tissues, which may explain the multiple effects of 20E as listed in Table 2 and the activity of some medicinal plants listed in Table 1. Thus, the potential of exploiting the anabolic effects of 20E to contribute to medical conditions and syndromes where declining muscle mass and performance are serious components (sarcopenia, cachexia, Duchenne muscular dystrophy) is being realized and the first clinical trials are being performed to validate the use of 20E as a drug for these conditions. Metabolic syndrome is a further area of immense interest where the anabolic, hypolipidemic and anti-diabetic effects of 20E may find application. Moreover, it has recently appeared that given its cellular target, 20E could represent an adequate molecule to prevent the appearance of severe forms of COVID-19, and a clinical trial is under way.

20E is an intriguing molecule which appears to have significant potential to contribute to human health, both nutritionally and in the combat against chronic diseases and conditions. Aspects, such as its low oral bioavailability, need improvement, and it may be that more effective analogues can be identified in the future, but for the moment 20E is leading the way.

## Figures and Tables

**Table 1:** Selected examples of ecdysteroid-rich medicinal plants and their traditional uses. The names of ecdysteroids are underlined (see www.ecdybase.org for structures, accessed on 1 April 2021). Note that it is not established that all the reported biological activities are due totally or partly to the presence of ecdysteroids.

Scientific Name	Plant Part	Major Constituents	Claimed Therapeutical Value	World Area	References
*Achyranthes bidentata*	root	20E, Inokosterone, polysaccharides	Anticancer, anti-inflammatory, diuretic, anti-osteoporotic	India, China Taiwan	[8,9]
*Achyranthes japonica*	root, leaf	Inokosterone, 20E, saponin, oleanolic acid, calcium oxalate	Antirheumatic, for amenorrhea, carbuncles, fever, dystocia, urinary ailments	Korea, Japan, China	[10]
*Ajuga bracteosa* *A. decumbens*	whole plant	20E, ajugasterone C ajugalactone, cyasterone, kiransin, β-sitosterol, cerotic acid, palmitic acid, luteolin	Antitussive, antipyretic, anti-inflammatory, antiphlogistic, antibacterial; treats bladder ailments, diarrhea, bronchitis	Taiwan	[11]
*Ajuga iva*	aerial parts	20E, cyasterone, ajugasterone C, apigenin, apigenin dihexoside, carvacrol	Diabetes, rheumatism, allergy, cancer, renal, metabolic disorders, digestive, cardiovascular, and respiratory disorders	Africa	[12,13]
*Ajuga turkestanica*	aerial parts	20E, turkesterone, cyasterone, iridoids	Weight deficiency, ulcers, burns, wound healing, heart protective, hair growth	Uzbekistan, Tadzhikistan	[14]
*Boerhaavia diffusa*	root, aerial parts	20E, flavonoids, rotenoids, punarnavoside	Immunomodulatory, anticancer, antidiabetic, anti-inflammatory, diuretic, hepatoprotective, antimicrobial, antifungal, anticonvulsant, antioxidant	Brazil, India, Iran, Angola, Ghana, Congo	[15]
*Cyathula prostrata*	leaf, root	20E, cyasterone, terpenoids, flavonols, tannins	Laxative, antitoxic, analgesic, alleviates flu, cough, rheumatism, dysentery, syphilis	Tropical Africa, China, Australia	[8,16,17]
*Cyanotis arachnoidea*	aerial parts, root	20E, ajugasterone C, poststerone, rubrosterone, daucosterol	Skin diseases, psoriasis, gastritis, tuberctulosis	China	[18]
*Diploclisia glaucescens*	stem, leaf	paristerone, 20E, capitasterone, oleanane glycosides	Rheumatism, snake venom, biliousness, venereal diseases	China	[19]
*Helleborus niger*	rhizomes, leaves	20E, polypodine B, bufadienolides,	Stomachic, tonic, analgesic, antirheumatic	Romania	[20]
*Microsorum membranifolium*	fronds	20E, ecdysone, 2-deoxy-20E, 2-deoxyecdysone, various conjugates	Asthma, purgative, antiemetic, healing of fractured bones	French Polynesia	[21]
*Paris polyphylla*	aerial parts	20E, calonysterone, steroidal saponins, luteolin, quercetin	Antibiotic, anti-inflammatory, liver cancer	Southwest China	[22]
*Pfaffia glomerata*	root	20E, pterosterone, polypodine B, ginsenosides	General stimulant, analgesic, anabolic, anti-inflammatory, immunostimulant, sedative, hypocholesterolemic	Brazil	[23]
*Podocarpus macrophyllus* var. *nakaii*	stem bark, leaf, root, fruit	20E, ponasterone A, makisterones, pinene, camphene, cadinene, podocarpene, kaurene	Antihelminthic, blood disorders; tonic for heart, kidneys, lungs, stomach	South Africa	[8,24]
*Polypodium vulgare*	rhizome	20E, polypodine B, polypodaurein, polypodosaponin, flavonoids	Expectorant, cough, pertussis, diuretic	Poland	[25]
*Rhaponticum carthamoides*	leaf, root	20E, makisterones, triterpenes, sesquiterpene lactones, phenolic acids, flavonoids, thiophenes, lignans	Tonic, roborant, adaptogenic, antidepressive, antiparasitic	Eastern Europe	[26,27]
*Serratula chinensis*	roots	20E glycosides, sphingolipids, cerebrosides	Pharyngitis, measles, anti-inflammatory, hypocholesterolemic, anti-cancer,	Southern China	[8,28]
*Sida rhombifolia*	root, seed	20E, ecdysone, ecdysteroid glycosides, cyclopropenoid fatty acids	Enteritis, hepatitis, flu, pneumonia, improves blood circulation, resolves phlegm, alleviates pain	India	[8,29]
*Tinospora cordifolia*	aerial parts	20E, polypodine B, alkaloids, diterpenoid lactones, sinapic acid	Anti-osteoporotic, anti-inflammatory, anti-stress, immuno-modulator, anti-spasmodic, chemo- and radio-protective, anti-anxiety, neuroprotective	India	[30,31]
*Vitex scabra*	bark, leaf	20E, turkesterone, khainaoside, syringaresinol	Astringent, antihelminthic, gastrointestinal disorders, wound healing, sexual enhancer	Thailand	[32]

**Table 2:** Summary of the literature concerning 20E’s pharmacological effects in mammals.

Effect	20E and/or Other Ecdysteroid Preparations
Anabolic (muscle)	[46,47,48,49,50,51,52,53,54]
Fat-reducing/Hypolipidaemic	[55,56,57,58,59,60,61,62]
Anti-diabetic	[56,62,63,64,65,66]
Anti-fibrotic	[67]
Anti-inflammatory	[68,69]
Anti-oxidant	[70]
Anti-thrombotic	[71,72]
Vasorelaxant	[73]
Hematopoiesis stimulation	[74]
Angiogenic	[75,76]
Cardioprotective	[62,77,78,79]
Neuromuscular protective	[80,81]
Neuroprotective	[81,82,83,84]
Liver protective	[85,86]
Lung protective	[69,72,87,88]
Kidney protective	[58,67,89]
Gastric protective	[90,91]
Bone, cartilage protective	[92,93,94,95,96,97,98,99]
Skin protective/repairing	[100,101,102]

**Table 3:** The effect of ^3^H-*Achyranthes bidentata* ecdysterone on the quantitative distribution of different tissues in different tracking phases of mice (mean, *n* = 5) expressed in μg g^−1^ (translated from Wu et al. [124]).

	5 min	10 min	30 min	1 h	3 h	6 h	12 h	24 h
Blood plasma	0.061	0.057	0.052	0.047	0.032	0.023	0.015	0.011
Urine	0.482	0.921	1.534	0.281	0.102	0.096	0.087	0.046
Feces	0.015	0.019	0.035	0.068	0.099	0.931	0.312	0.041
Bile	0.421	0.456	1.042	0.901	0.301	0.209	0.198	0.094
Liver	0.312	0.251	0.213	0.112	0.078	0.061	0.056	0.046
Heart	0.062	0.052	0.035	0.033	0.031	0.029	0.023	0.022
Spleen	0.041	0.026	0.024	0.018	0.023	0.027	0.032	0.027
Lungs	0.116	0.069	0.057	0.053	0.049	0.045	0.036	0.028
Kidney	0.137	0.123	0.098	0.071	0.055	0.043	0.037	0.032
Adrenals	0.184	0.139	0.098	0.081	0.073	0.065	0.052	0.043
Testis	0.048	0.036	0.029	0.026	0.023	0.021	0.019	0.017
Skeletal muscle	0.021	0.023	0.028	0.017	0.015	0.013	0.011	0.010
Spinal cord	0.019	0.028	0.067	0.041	0.033	0.024	0.020	0.018
Brain	0.013	0.014	0.015	0.013	0.012	0.01	0.008	0.007
Small intestine	0.254	0.139	0.094	0.083	0.076	0.061	0.05	0.033
Stomach	0.034	0.047	0.075	0.061	0.053	0.041	0.032	0.023
Large intestine	0.011	0.023	0.029	0.031	0.035	0.048	0.038	0.023
Bladder	0.013	0.024	0.038	0.026	0.023	0.018	0.015	0.013

**Table 4:** Kinetic parameters of BIO101 (=97% pure 20E) after a single oral administration (*n* = 6, fasted). Values in parentheses indicate the SEM (C_max_, AUC) or the range (t_max_).

B1O101 PK Parameter (Unit)	100 mg	350 mg	700 mg	1400 mg
Cmax (ng/mL)	141 (16.6)	317 (37.9)	399 (24.7)	710 (20.2)
tmax (h)	2.03 (1.00–3.02)	3.00 (1. 05–4.00)	3.00 (2.00–4.02)	3.50 (2.00–4.02)
AUC0-t (ng·h/mL)	767 (31.1)	1924 (40.1)	2578 (22.9)	4148 (15.9)

**Table 5:** Non-clinical regulatory requirements and submission dossier for clinical trial approval.

Non-Clinical Activities	Studies	Clinical Trial		
		Non Medicinal Drug Trial	Phase 1	Phase 2
Regulatory requirements				
Absorption	Pharmacokinetics	NA	✓	✓
	Toxicokinetics	NA	✓	✓
	Transporters (Caco-2 cells)	NA	✓	✓
Metabolism	Microsomal metabolism	NA	NA	✓
	ADME ^$^ study	NA	NA	✓
	CYP inhibition/induction	NA	✓	✓
	Metabolite identification	NA	✓	✓
Toxicology	Phototoxicity	NA	✓	✓
	Genotoxicity tests (Ames; micronuclei)	NA	✓	✓
	Repeated toxicology studies	NA	✓	✓
	Micronuclei tests	NA	✓	✓
Distribution	Red blood cell partitioning	NA	✓	✓
	Plasma protein binding	NA	✓	✓
Pharmacology	In vitro studies	✓	✓	✓
	In vivo studies	✓	✓	✓
Safety pharmacology	Cardiovascular system	NA	✓	✓
	Central nervous system and respiratory function	NA	✓	✓
**Documents required for submission dossier**				
Investigational Medicinal Product Dossier (IMPD)		NA	✓	✓
Investigation Brochure (IB)		NA	✓	✓
Technical Product Dossier		✓	NA	NA
Literature related to the product		✓	NA	NA
Clinical Trial Protocol		✓	✓	✓
Informed consent		✓	✓	✓
Investigative New Drug (IND) Package for the FDA		NA	✓	✓

NA: not-applicable; ✓: required; ^$^ “adsorption, distribution, metabolism and excretion”.

**Table 6:** Drugability scores for ecdysteroids.

Compound	MW	LogP	PSA (Å^2^)	Rotatable Bonds	H-Acceptors	H-Donors	TL Score
20E	480.30	1.36	138.45	5	7	6	3
20,26E	496.64	0.35	158.67	6	8	7	4
14d20E	464.64	2.30	118.21	5	6	5	1
6αOH20E	482.66	1.54	141.60	5	7	7	4
6αOH14d20E	466.66	2.49	121.37	5	6	6	3
Post	362.46	1.03	94.83	1	5	3	0
14dPost	346.47	1.97	74.60	1	4	2	0
6αOHPost	364.48	1.22	97.98	1	5	4	0
6αOH14dPost	348.48	2.16	77.75	1	4	3	0

The canonical isomeric SMILES string was created for each ecdysteroid at http://endocrinedisruptome.ki.si (accessed on 15 July 2019), which allows full stereochemical specification of the structure (including C-5 and C-9), and pasted into the Molinspiration calculator (www.molinspiration.com, accessed on 15 July 2019) to obtain values for the above parameters. Scoring was as follows: MW: <400 = 0; 400–500 = 1; >500 = 2; Calculated LogP (calculated by fragment summation method): <3 = 0; 3–5 = 1; >5 = 2; Polar surface area PSA (Å^2^): <120 = 0; 120–140 = 1; >140 = 2; Rotatable bonds (excluding C-O-H): <7 = 0; 8–10 = 1; >11 = 2; Number of NH + OH (H-bond donors): ≤5 = 0; >5 = 1; Number of N+O (H-bond acceptors): ≤10 = 0; >10 = 1.

**Table 7:** Clinical studies on humans using pure 20E.

Aim	Age	N	Dose	Duration	Output	Ref
Sexual disadaptation	27–61	93 20F, 73M	7.5–10 mg/day	1 month	Improvement of libido and sexual activity in 75% of patients	[165]
Chonic glomerulonephritis	35 ± 7	35	15 mg/day	10 days	Improvement of kidney function and of microcirculation	[166]
Sexual function	N/A, M	60	5 mg, 2×/day	30 days	Improved sperm quality and copulative function in patients with disturbed spermatogenesis as a complication of urologic diseases Improvement of sexual function during recovery from myocardial infarction	[158]
	40–60 M	48	5 mg 3×/day			
Giardiasis	N/A	35	5 mg 3× or 4×/day	10 days	Parasite elimination in 68.7% of patients	[167]
Hepatitis	N/A	N/A	5 mg b.i.d.	30 days	In case of hepatitis B, improvement of liver state	[159]
Lambliasis	N/A	N/A	5 mg 4×/day	10 days	Therapy eliminated most parasites within 10 days	[168]
Hymenolepiasis	N/A	22	5 mg 3×/day	2 weeks	Reduction in symptoms and parasitological efficacy of 36.4%	[169]
Metabolic syndrome	Overweight	39	2 × 50 mg/day	3 months?	Reduction in body weight (−1.3%) waist circumference (−3.2%), body fat (−7.6%), C-reactive protein (−38%), total cholesterol (−17%), triglycerides (−37%), muscle increase (+2.9%)	[170]
Menopause disorders	Overweight	N/A	100 or 200 mg/day	3 months	Prevention of metabolic syndrome and osteoporosis, reduction in body weight, reduction in plasma cholesterol and CRP; proposed for hormone replacement therapy	[171,172]
Sarcopenia	≥65	231	175/350 mg b.i.d.	6–9 months	*Expected*: change from baseline of gait speed (400MW test), appendicular lean mass and handgrip strength	[173]
Metabolic syndrome	>18	64 test 28 Control	40 or 90 mg/day	3–6–9 months	Reductions in body mass, proportion of body fat, waist cicumference and hsCRP, retention of muscle mass	[174]
Prediabetes	30–60	34	300 mg/day	12 weeks	*Expected*: changes in micronuclei, reduction in fasted glycemia, glycated hemoglobin	[175]
ARDS in COVID 19	≥55	310	350 mg b.i.d.	28 days	*Expected*: Prevention of respiratory deterioration in severe COVID-19 patients	[176]
Celiac disease	Children 3–14	25	2.5 mg/kg/day	14 days	Reduction in symptoms Improvement of energy metabolism	[177]

M: male; F: female; b.i.d.: bis in die (twice a day); N/A: information not available.

## Data Availability

Not applicable.

## Abbreviations

E	ecdysone
20E	20-hydroxyecdysone
14d20E	14-deoxy-20-hydroxyecdysone
Post	poststerone
14dPost	14-deoxypoststerone
3-epi-Post	3-*epi*-poststerone
16αOHPost	16α-hydroxypoststerone
20R/SPost	20-dihydropoststerone
21OHPost	21-hydroxypoststerone
20,26E	20,26-dihydroxyecdysone
PolB	polypodine B

## References

[1] Koolman J. (1989). Ecdysone: From Chemistry of Mode of Action.

[2] Dinan L., Savchenko T., Whiting P. (2001). On the distribution of phytoecdysteroids in plants. Cell Mol. Life Sci.

[3] Dinan L., Harmatha J., Volodin V., Lafont R., Smagghe G. (2009). Phytoecdysteroids: Diversity, biosynthesis and distribution. Ecdysone: Structures and Functions.

[4] Lafont R., Harmatha J., Marion-Poll F., Dinan L., Wilson I.D. (2002). The Ecdysone Handbook. http://ecdybase.org/.

[5] Dinan L., Mamadalieva N., Lafont R., Xiao J., Sarker S.D., Asakawa Y. (2020). Dietary Phytoecdysteroids. Handbook of Dietary Phytochemicals.

[6] Kametani T., Tsubuki M., Koolman J. (1989). Strategies for the synthesis of ecdysteroids. Ecdysone. From Chemistry to Mode of Action.

[7] Sláma K., Lafont R. (1995). Insect hormones—ecdysteroids: Their presence and action in vertebrates. Eur J. Entomol.

[8] Li T.S.C. (2006). Taiwanese Native Medicinal Plants.

[9] He X., Wang X., Fang J., Chang Y., Ning N., Guo H., Huang L., Huang X. (2017). The genus *Achyranthes*: A review on traditional uses, phytochemistry, and pharmacological activities. J. Ethnopharmacol..

[10] Choi H.S., Lee M.J., Na M.S., Lee M.Y., Choi D. (2019). Antioxidant properties of *Achyranthis* radix extract in rats. J. Ind. Eng. Chem..

[11] Hsieh W.T., Liu Y.T., Lin W.C. (2011). Anti-inflammatory properties of *Ajuga bracteosa* in vivo and in vitro study and their effects on mouse models of liver fibrosis. J. Ethnopharmacol..

[12] Israili Z.H., Lyoussi B. (2009). Ethnopharmacology of the plants of genus *Ajuga*. Pak. J. Pharm Sci.

[13] Bouyahya A., El Omari N., Elmenyiy N., Guaouguaou F.E., Balahbid A., El-Shazly M., Chamkhi I. (2020). Ethnopharmacological use, phytochemistry, pharmacology, and toxicology of *Ajuga iva* (L.) Schreb. J. Ethnopharmacol..

[14] Cheng D.M., Yousef G.G., Grace M.H., Rogers R.B., Gorelick-Feldman J., Raskin I., Lila M.A. (2008). In vitro production of metabolism-enhancing phytoecdysteroids from *Ajuga turkestanica*. Plant Cell Tiss Organ Cult..

[15] Patil K.S., Bhaising S.R. (2016). Ethnomedicinal uses, phytochemistry and pharmacological properties of the genus *Boerhaavia*. J. Ethnopharmacol..

[16] Ibrahim B., Sowemimo A., van Rooyen A., Van de Venter M. (2012). Antiinflammatory, analgesic and antioxidant activities of *Cyathula prostrata* (Linn.) Blume (Amaranthaceae). J. Ethnopharmacol..

[17] Ajuogu P.K., Ere R., Nodu M.B., Nwachikwu C.U., Lgbere O.O. (2020). The influence of graded levels of *Cyathula prostrata* (Linn.) Blume on semen quality characteristics of adult New Zealand white bucks. Transl. Anim. Sci..

[18] Lee S., Xiao C., Pei S. (2008). Ethnobotanical survey of medicinal plants at periodic market of Honhe Prefecture in Yunnan Province, SW China. J. Ethnopharmacol..

[19] Fang L., Li J., Zhou J., Wang X., Guo L. (2017). Isolation and purification of three ecdysteroids from the stems of *Diploclisia glaucescens* by high-speed countercurrent chromatography and their anti-inflammatory activities *in vitro*. Molecules.

[20] Schink M., Garcia-Käufer M., Bertrams J., Duckstein S.M., Müller M.B., Huber R., Stintzing F.C., Gründemann C. (2015). Differential cytotoxic properties of *Helleborus niger* L. on tumour and immunocompetent cells. J. Ethnopharmacol..

[21] Ho H., Teai T., Bianchini J.P., Lafont R., Raharivelomanana P., Fernández H. (2011). Ferns: From traditional uses to pharmacological development, chemical identification of active principles. Working with Ferns: Issues and Applications.

[22] Zhu L., Tan J., Wang B., Guan L., Liu Y., Zheng C. (2011). In vitro antitumor activity and antifungal activity of Pennogenin steroidal saponins from *Paris polyphylla* var. yunnanensis. Iranian J. Pharm. Res..

[23] Franco R.R., de Almeida Takata L., Chagas K., Justino A.B., Saraiva A.L., Goulart L.R., de Melo Rodrigues Ávila V., Otoni W.C., Espindola F.A., da Silva C.R. (2021). A 20-hydroxyecdysone-enriched fraction from *Pfaffia glomerata* (Spreng.) pedersen roots alleviates stress, anxiety, and depression in mice. J. Ethnopharmacol..

[24] Abdillahi H.S., Finnie J.F., Van Staden J. (2011). Anti-inflammatory, antioxidant, anti-tyrosinase and phenolic contents of four *Podocarpus* species used in traditional medicine in South Africa. J. Ethnopharmacol..

[25] Gleńsk M., Dudek M.K., Ciach M., Wlodarczyk M. (2019). Isolation and structural determination of flavan-3-ol derivatives from the *Polypodium vulgare* L. rhizomes water extract. Nat. Prod. Res..

[26] Kokoska L., Janikova D. (2009). Chemistry and pharmacology of *Rhaponticum carthamoides*: A review. Phytochemistry.

[27] Shikov A.N., Narkevich I.A., Flisyuk E.V., Luzhanin V.G., Pozharitskaya O.N. (2021). Medicinal plants from the 14th edition of the Russia, Pharmacopoeia, recent updates. J. Ethnopharmacol..

[28] Zhang Z.-Y., Yang W.-Q., Fan C.-L., Zhao H.-N., Huang X.-J., Wang Y., Ye W.-C. (2017). New ecdysteroid and ecdysteroid glycosides from the roots of Serratula chinensis. J. Asian Nat. Prod. Res..

[29] Dinda B., Das N., Dinda S., Dinda M., SilSarma I. (2015). The genus *Sida,* L. A traditional medicine: Its ethnopharmacological phytochemical and pharmacological data for commercial exploitation in herbal drug industry. J. Ethnopharmacol..

[30] Bala M., Pratap K., Verma P.K., Singh B., Padwad Y. (2015). Validation of ethnopharmacological potential of *Tinospora cordifolia* for anticancer and immunomodulatory activities and quantification of bioactive molecules by HPTLC. J. Ethnopharmacol..

[31] Sharma B., Dutt V., Kaur N., Mittal A., Dabur R. (2020). *Tinospora cordifolia* protects from skeletal muscle atrophyby alleviating oxidative stress and inflammation induced by sciatic denervation. J. Ethnopharmacol..

[32] Suksamrarn A., Kumpun S., Yingyongnarongkul B. (2002). Ecdysteroids from *Vitex scabra* bark. J. Nat. Prod..

[33] Báthori M. (2002). Phytoecdysteroids effects on mammalians, isolation and analysis. Mini Rev. Med. Chem..

[34] Lafont R., Dinan L. (2003). Practical uses for ecdysteroids in mammals including humans: An update. J. Insect. Sci..

[35] Báthori M., Pongrácz Z. (2005). Phytoecdysteroids—From isolation to their effects on Humans. Curr. Med. Chem..

[36] Dinan L., Lafont R. (2006). Effects and application of arthropod steroid hormones (ecdysteroids) in mammals. J. Endocrinol..

[37] Zou D., Cao L., Chen Q. (2008). Advances in pharmacological research on ecdysteroids. Zhongguo Xinyao Zazhi.

[38] Báthori M., Tóth N., Hunyadi A., Márki A., Zador E. (2008). Phytoecdysteroids and anabolic-androgenic steroids—Structure and effects on Humans. Curr. Med. Chem..

[39] Lafont R., Dinan L., Smagghe G. (2009). Innovative and future applications of ecdysteroids. Ecdysone: Structures and Functions.

[40] Cahliková L., Macáková K., Chlebek J., Hošt’álková A., Kulkhánková A., Opletal L. (2011). Ecdysterone and its activity on some degenerative diseases. Nat. Prod. Commun..

[41] Lafont R. (2012). Recent progress in ecdysteroid pharmacology. Theor Appl. Ecol..

[42] Laekeman G., Vlietinck A., Ramawat K.G., Mérillon J.M. (2013). Phytoecdysteroids: Phytochemistry and pharmacological activity. Natural Products.

[43] Bajguz A., Bakala I., Talarek M., Atta-ur-Rahman F.R.S. (2015). Ecdysteroids in plants and their pharmacological effects in vertebrates and Humans. Studies in Natural Product Chemistry.

[44] Dinan L., Lafont R. (2015). Phytoecdysteroids occurrence, distribution, biosynthesis, metabolism, mode of action and applications: Developments from 2005 to 2015. Pharm Bull. J. (Kazakhstan).

[45] Das N., Mishra S.K., Bishayee A., Ali E.S., Bishayee A. (2020). The phytochemical, biological, and medicinal attributes of phytoecdysteroids: An updated review. Acta Pharm. Sinica B.

[46] Chermnykh N.S., Shimanovsky N.L., Shutko G.V., Syrov V.N. (1988). Effects of methandrostenolone and ecdysterone on physical endurance of animals and protein metabolism in the skeletal muscles. Farmakol. Toksikol..

[47] Tóth N., Szabó A., Kacsala P., Héger J., Zádor E. (2008). 20-Hydroxyecdysone increases fiber size in a muscle-specific fashion in rat. Phytomedicine.

[48] Syrov V.N. (2000). Comparative experimental investigations of the anabolic activity of ecdysteroids and steranabols. Pharm. Chem. J..

[49] Gorelick-Feldman J., MacLean D., Ilic N., Poulev A., Lila M.A., Cheng D., Raskin I. (2008). Phytoecdysteroids increase protein synthesis in skeletal muscle cells. J. Agric. Food Chem..

[50] Gorelick-Feldman J., Cohick W., Raskin I. (2010). Ecdysteroids elicit a rapid Ca^2+^ flux leading to Akt activation and increased protein synthesis in skeletal muscle cells. Steroids.

[51] Goreclick-Feldman J.I. (2009). Phytoecdysteroids: Understanding their Anabolic Activity. Ph.D. Thesis.

[52] Lawrence M.M. (2012). *Ajuga turkestanica* as a Countermeasure against Sarcopenia and Dynapenia. Master’s Thesis.

[53] Zubeldia J.M., Hernández-Santana A., Jiménez-del-Rio M., Pérez-López V., Pérez-Machín R., Garcia-Castellano J.M. (2012). In vitro characterization of the efficacy and safety profile of a proprietary *Ajuga turkestanica* extract. Chinese Med..

[54] Parr M.K., Zhao P., Haupt O., Tchoukouegno Ngueu S., Hengevoss J., Fritzemeier K.H., Piechotta M., Schlörer N., Muhn P., Zheng W.Y. (2014). Estrogen receptor beta is involved in skeletal muscle hypertrophy induced by the phytoecdysteroid ecdysterone. Mol. Nutr. Food Res..

[55] Syrov V.N., Khushbaktova Z.A., Abzalova M.K., Sultanov M.B. (1983). On the hypolipidemic and antiatherosclerotic action of phytoecdysteroids. Doklady Akademii Nauk Uzbekistan SSR.

[56] Kizelsztein P., Govorko D., Komarnytsky S., Evans A., Wang Z., Cefalu W.T., Raskin I. (2009). 20-Hydroxyecdysone decreases weight and hyperglycemia in a diet-induced obesity mice model. Am. J. Physiol. Endocrinol. Metab..

[57] Seidlova-Wuttke D., Ehrhardt C., Wuttke W. (2010). Metabolic effects of 20-OH-ecdysone in ovariectomized rats. J. Steroid Biochem Mol. Biol.

[58] Syrov V.N., Khushbaktova Z.A., Nabiev A.N. (1992). An experimental study of the hepatoprotective properties of phytoecdysteroids and nerobol in carbon tetrachloride—Induced liver injury. Eksp. Klin. Farmakol..

[59] Foucault A.-S., Mathé V., Lafont R., Even P., Dioh W., Veillet S., Tomé D., Huneau D., Hermier D., Quignard-Boulangé A. (2012). Quinoa extract enriched in 20-hydroxyecdysone protects mice from diet-induced obesity and modulates adipokines expression. Obesity.

[60] Foucault A.-S., Even P., Lafont R., DIoh W., Veillet S., Tomé D., Huneau J.F., Hermier D., Quignard-Boulangé A. (2014). Quinoa extract enriched in 20-hydroxyecdysone affects energy homeostasis and intestinal fat absorption in mice fed a high-fat diet. Physiol. Behav..

[61] Agoun H., Semiane N., Mallek A., Bellahreche Z., Hammadi S., Madjerab M., Abdlalli M., Khalkhal A., Dahmani Y. (2019). High-carbohydrate diet-induced metabolic disorders in *Gerbillus tarabuli* (a new model of non-alcoholic fatty liver disease). Protective effects of 20-hydroxyecdysone. Arch. Physiol. Biochem..

[62] Buniam J., Chukijrungroat N., Rattanavichit Y., Surapongchai J., Weerachayaphom J., Bupha-Intr T., Saengsirisuwan V. (2020). 20-Hydroxyecdysone ameliorates metabolic and cardiovascular dysfunction in high-fat-high-fructose-fed ovariectomized rats. BMC Complement. Med. Ther..

[63] Yoshida T., Otaka T., Uchiyama M., Ogawa S. (1971). Effect of ecdysterone on hyperglycemia in experimental animals. Biochem. Pharmacol..

[64] Sundaram R., Naresh R., Shanthi P., Sachdanandam P. (2012). Efficacy of 20-OH-ecdysone on hepatic key enzymes of carbohydrate metabolism in streptozotocin induced diabetic rats. Phytomedicine.

[65] Sundaram R., Naresh R., Shanthi P., Sachdanandam P. (2012). Ameliorative effect of 20-OH ecdysone on streptozotocin induced oxidative stress and β-cell damage in experimental hyperglycemic rats. Process. Biochem..

[66] Chen L., Zheng S., Huang M., Ma X., Yang J., Deng S., Huang Y., Wen Y., Yang X. (2017). β-edysterone from *Cyanotis arachnoidea* exerts hypoglycemic effects through activation of IRS-1/Akt/GLUT4 and IRS-1/Akt/GLUT2 signal pathways in KK-Ay mice. J. Funct. Foods.

[67] Hung T.J., Chen W.M., Liu S.F., Liao T.N., Lee T.C., Chuang L.Y., Guh J.Y., Hung C.Y., Hung H.J., Chen P.Y. (2012). 20-Hydroxyecdysone attenuates TGF-β1-induced renal cellular fibrosis in proximal tubule cells. J. Diabetes Complicat..

[68] Kurmukov A.G., Syrov V.N. (1988). Anti-inflammatory properties of ecdysterone. Meditsinskii Zhurnal Uzb..

[69] Song G., Xia X.C., Zhang K., Yu R., Li B., Li M., Yu X., Zhang J., Xue S. (2019). Protective effect of 20-hydroxy-ecdysterone against lipopolysaccharides-induced acute lung injury in mice. J. Pharm. Drug Res..

[70] Cai Y.J., Dai J.Q., Fang J.G., Ma L.P., Hou L.F., Yang L., Liu Z.L. (2002). Antioxidative and free radical scavenging effects of ecdysteroids from *Serratula strangulata*. Can. J. Physiol. Pharmacol..

[71] Azizov A.P. (1997). Effects of eleutherococcus, elton, leuzea, and leveton on the blood coagulation system during training in athletes. Eksp Klin Farmakol.

[72] Xia X.C., Xue S.P., Wang X.Y., Liu R. (2016). Effects of 20-hydroxyecdysone on expression of inflammatory cytokines in acute lung injury mice. Mod. Prev. Med..

[73] Dongmo A.B., Nkeng-Efouet P.A., Devkota K.P., Wegener J.W., Sewald N., Wagner H., Vierling W. (2014). Tetra-acetylajugasterone a new constituent of *Vitex cienkowskii* with vasorelaxant activity. Phytomed.

[74] Syrov V.N., Nasyrova S.S., Khushbaktova Z.A. (1997). The results of experimental study of phytoecdysteroids as erythropoiesis stimulators in laboratory animals. Eksperimental’naia i Klin. Farmakol..

[75] Chen Z., Zhu G., Zhang J.H., Liu Z., Tang W., Feng H. (2008). Ecdysterone-sensitive smooth muscle cell proliferation stimulated by conditioned medium of endothelial cells cultured with bloody cerebrospinal fluid. Acta Neurochir Suppl..

[76] Luo H., Yi B., Fan W., Chen K., Gui L., Chen Z., Li L., Feng H., Chi L. (2011). Enhanced angiogenesis and astrocyte activation by ecdysterone treatment in a focal cerebral ischemia rat model. Acta Neurochirurgica Suppl..

[77] Kurmukov A.G., Ermishina O.A. (1991). The effect of ecdysterone on experimental arrhythmias and changes in the hemodynamics and myocardial contractility induced by coronary artery occlusion. Farmakol. Toksikol..

[78] Korkach Y.P., Kotsyuruba A.V., Psryslazhnam O.D., Mohyl’nyts’ka L.D., Sahach V.F. (2007). NO-dependent mechanisms of ecdysterone protective action on the heart and vessels in streptozotocin-induced diabetes mellitus in rats. Fiziol. Zhurnal.

[79] Xia X., Zhang Q., Liang G., Lu S., Yang Y., Tian Y. (2013). Role of 20-hydroxyecdysone in protecting rats against diabetic cardiomyopathy. Chln J. Geriatr Heart Brain Vessel Dis..

[80] Dilda P., Latil M., Didry-Barca B., On S., Serova M., Veillet S., Lafont R. (2019). BIO101 demonstrates combined beneficial effects on skeletal muscle and respiratory functions in a mouse model of Duchenne muscular dystrophy. World Muscle Society WMS 2019, Copenhagen, Denmark (1-5/10/2019). Neuromuscul. Disord..

[81] Latil M., Bézier C., Cottin S., Lafont R., Veillet S., Dilda P., Charbonnier F., Biondi O. (2019). BIO101 demonstrates combined beneficial effects on muscle and motor neurons in a mouse model of severe spinal muscular atrophy. World Muscle Society WMS 2019, Copenhagen, (1-5/10/2019). Neuromuscul. Disord..

[82] Luo H., Luo C., Zhang Y., Chi L., Li L., Chen K. (2009). Effect of ecdysterone on injury of lipid peroxidation following focal cerebral ischemia in rats. Zhongguo Yaoye.

[83] Liu Z., Chen Y., Chen Z., Tang W., Zhu G., Wang X., Feng H. (2011). Effect of ecdysterone on the nervous lesions of rabbits acquired after subarachnoid hemorrhage. Med. J. Chin. People’s Lib. Army.

[84] Hu J., Luo C.X., Chu W.H., Shen Y.A., Qian Z.-M., Zhu G., Yu Y.B., Feng H. (2012). 20-Hydroxyecdysone protects against oxidative stress-induced neuronal injury by scavenging free radicals and modulating NF-kB and JNK pathways. PLoS ONE.

[85] Shakhmurova G.A., Khushbaktova Z.A., Syrov V.N. (2010). Estimation of hepatoprotective and immunocorrecting effects of the sum of phytoecdysteroids from *Silene viridiflora* in experimental animals treated with tetrachlormethan. O’zbekiston Biol. J..

[86] Xia X., Zhang Q., Wang Z., Gui G., Liang G., Liu R. (2013). Protective effect of 20-hydroxyecdysone on diabetic hepathopathy of rats. Xiandai Yufang Yixue.

[87] Wu X., Jiang Y., Fan S., Wang R., Xiang M., Niu H., Li T. (1998). Effects of ecdysterone on rat lung reperfusion injury. Chin. Pharm. Bull..

[88] Li J., Wu X., Zhang J., Wu X., Gao D., Shen T., Gu C. (2013). Effect of ecdysterone on the expression of toll-like receptor 4 and surfactant protein A in lung tissue of rats with acute lung injury. Infect. Inflamm. Repair.

[89] Zou D., Xu Z., Cao L., Chen Q. (2010). Effects of ecdysterone on early stage diabetic nephropathy in streptozotocin-induced diabetic rats. Chin. J. New Drugs Clin. Remedies.

[90] Shakhmurova G.A., Syrov V.N., Khushbaktova Z.A. (2010). Immunomodulating and antistress activity of ecdysterone and turkesterone under immobilization-induced stress conditions in mice. Pharm. Chem. J..

[91] Zhou Y., Wu X., Liao J., Wu C., Zhang Y., Zhang Z. (2010). Effect of ecdysterone on the healing of gastric ulcer in model rats. China Pharm..

[92] Gao L., Cai G., Shi X. (2009). β-Ecdysterone induces osteogenic differentiation in mouse mesenchymal stem cells and relieves osteoporosis. Biol. Pharm. Bull..

[93] Kapur P., Wuttke W., Jarry H., Seidlova-Wuttke D. (2010). Beneficial effects of β-ecdysone on the joint, epiphyseal cartilage tissue and trabecular bone in ovariectomized rats. Phytomed.

[94] Seidlova-Wuttke D., Christel D., Kapur P., Nguyen B.T., Jarry H., Wuttke W. (2010). β-Ecdysone has bone protective but no estrogenic effects in ovariectomized rats. Phytomedicine.

[95] Dai W., Zhang H., Zhong Z.A., Jiang L., Chen H., Lay Y.A., Kot A., Ritchie R.O., Lane N.E., Yao W. (2015). β-Ecdysone augments peak bone mass in mice of both sexes. Clin. Orthop Relat Res..

[96] Dai W., Jiang L., Evan Lay Y.-A., Chen H., Jin G., Zhang H., Kot A., Ritchie R.O., Lane N.E., Yao W. (2015). Prevention of glucocorticoid induced bone changes with beta-ecdysone. Bone.

[97] Wang G., Zhang X., Zhang W., Xia L. (2015). Protective effect of ecdysterone on rabbits chondrocytes that is injured by lipopolysaccharide. Tianjin Med J..

[98] Wen F., Yu J., He C.J., Zhang Z.W., Yang A.F. (2019). β-ecdysterone protects against apoptosis by promoting autophagy in nucleus pulposus cells and ameliorates disc degeneration. Mol. Med. Rep..

[99] Zhao Q., Wang Z., He J. (2021). Effect of β-ecdysterone on the proliferation, differentiation and apoptosis of rat osteoblasts induced by high glucose. Chin. J. Clin. Pharmacol..

[100] Detmar M., Dumas M., Bonté F., Meybeck A., Orfanos C.E. (1994). Effects of ecdysterone on the differentiation of normal keratinocytes *in vitro*. Eur. J. Dermatol..

[101] Zhegn G.Y., Wu X., Li Y.L., Zfang J.H., Wang W.J. (2008). Preparation and dose-effect analysis of ecdysterone cream for promoting wound healing. J. Southern Med. Univ..

[102] Ehrhardt C., Wessels J.T., Wuttke W., Seidlova-Wuttke D. (2011). The effects of 20-hydroxyecdysone and 17β-estradiol on the skin of ovariectomized rats. Menopause.

[103] Dilda P., Foucault A.S., Serova M., On S., Raynal S., Veillet S., Dioh W., Lafont R. (2016). BIO101, a drug candidate targeting Mas receptor for the treatment of age-reated muscle degeneration. From molecular target identification to clinical development. J. Cachexia Sarcopenia Muscle.

[104] Antoshechkin A.G. (2016). Selective plant extracts and their combination as nutritional therapeutic remedies. J. Nutr Ther..

[105] Syrov V.N., Kurmukov A.G. (1967). Anabolic activity of phytoecdysone—ecdysterone—isolated from *Rhaponticum carthamoides* (Will.) Illjin. Farmakol. Toksikol..

[106] Kholodova I.D., Tugai V.A., Zimina V.P. (1997). Effect of vitamin D_3_ and 20-hydroxyecdysone on the content of ATP, creatine phosphate, carnosine and Ca^2+^ in skeletal muscles. Ukr. Biokhimicheskii Zhurnal.

[107] Lupien P.J., Hinse C., Chaudhary K.D. (1969). Ecdysterone as a hypoholesterolemic agent. Arch. Int. Physiol. Biochim..

[108] Catalan R.E., Martinez A.M., Aragones M.D., Miguel B.G., Robles A., Godoy J.E. (1985). Alterations in rat lipid metabolism following ecdysterone treatment. Comp. Biochem. Physiol..

[109] Matsuda H., Kawaba T., Yamamoto Y., Ogawa S. (1974). Effect of ecdysterone on experimental atherosclerosis in rabbits. Nippon Yakurigaku Zasshi.

[110] Catalan R.E., Aragones M.D., Godoy J.E., Martinez A.M. (1984). Ecdysterone induces acetylcholinesterase in mammalian brain. Comp. Biochem. Physiol..

[111] Chaudhary K.D., Lupien P.J., Hinse C. (1969). Effect of ecdysone on glutamic decarboxylase in rat brain. Experientia.

[112] Chiang H.C., Wang J.J., Wu R.T. (1992). Immunomodulating effects of the hydrolysis products of formosamin C and β-ecdysone from *Paris formosana* Hayata. Anticancer Res..

[113] Lafont R., Raynal S., Serova M., Didry-Barca B., Guibout L., Dinan L., Latil M., Dilda P.J., Dioh W., Veillet S. (2020). 20-Hydroxyecdysone activates the protective arm of the renin angiotensin system via Mas receptor. bioRxiv.

[114] Raynal S., Foucault A.-S., Ben Massoud S., Dioh W., Lafont R., Veillet S. (2015). BIO101, a drug candidate targeting sarcopenic obesity through MAS receptor activation. J. Cachexia Sarcopenia Muscle.

[115] Yoshida T., Galvez S., Tiwari S., Rezk B.M., Semprun-Prieto L., Higashi Y., Sukhanov S., Yablonka-Reuveni Z., Delafontaine P. (2013). Angiotensin II inhibits satellite cell proliferation and prevents skeletal muscle regeneration. J. Biol. Chem..

[116] Band M.M., Sumukadas D., Struthers A.D., Avenell A., Donnan P.T., Kemp P.R., Smith K.T., Hume C.L., Hapca A., Witham M.D. (2018). Leucine and ACE inhibitors as therapies for sarcopenia (LACE trial): Study protocol for a randomised controlled trial. Trials.

[117] Höcht C., Mayer M., Taira C.A. (2009). Therapeutic perspectives of angiotensin-(1-7) in the treatment of cardiovascular diseases. Open Pharmacol. J..

[118] Parr M.K., Botré F., Naß A., Hengevoss J., Diel P., Wolber G. (2015). Ecdysteroids: A novel class of anabolic agents?. Biol. Sport.

[119] Schreihofer D.A., Duong P., Cunningham R.L. (2018). N-terminal truncations in sex steroid receptors and rapid steroid actions. Steroids.

[120] Sobrino A., Vallejo S., Novella S., Lázaro-Franco M., Mompeón A., Bueno-Betí C., Walther T., Sánchez-Ferrer C., Peiró C. (2017). Mas receptor is involved in the estrogen-receptor induced nitric oxide-dependent vasorelaxation. Biochem. Pharmacol..

[121] Ogawa S., Nishimoto N., Matsuda H., Burdette W.J. (1974). Pharmacology of ecdysones un vertebrates. Invertebrate Endocrinology and Hormonal Heterophylly.

[122] Hikino H., Oizumi Y., Takemoto T. (1972). Absorption, distribution, metabolism, and excretion of insect-metamorphosing hormone ecdysterone in mice. Chem. Pharm. Bull. (Tokyo).

[123] Lafont R., Girault J.-P., Kerb U. (1988). Excretion and metabolism of injected ecdysone in the white mouse. Biochem. Pharmacol..

[124] Wu M., Zhao S., Ren L., Wang R., Bai X., Han H., Li B., Chen H. (2011). Research on the relationship between tissue quantitative distribution of ^3^H-*Achyranthes bidentata* ecdysterone and channel-tropism of herbal drugs in mice. China J. Chin. Mater. Med..

[125] Girault J.-P., Lafont R., Kerb U. (1988). Ecdysone catabolism in the white mouse. Drug Metab. Dispos..

[126] Kumpun S., Girault J.-P., Dinan L., Blais C., Maria A., Dauphin-Villemant C., Yingyongnarongkul B., Suksamrarn A., Lafont R. (2011). The metabolism of 20-hydroxyecdysone in mice: Relevance to pharmacological effects and gene switch applications of ecdysteroids. J. Steroid Biochem. Mol. Biol..

[127] Dinan L., Balducci C., Guibout L., Foucault A.S., Bakrim A., Kumpun S., Girault J.-P., Tourette C., Dioh W., Dilda P.J. (2021). Ecdysteroid metabolism in mammals: The fate of ingested 20-hydroxyecdysone in mice and rats. J. Steroid Biochem. Mol. Biol..

[128] Balducci C., Dinan L., Guibout L., Foucault A.S., Carbonne C., Durand J.-D., Caradeux C., Bertho G., Girault J.-P., Lafont R. (2021). The complex metabolism of poststerone in male rats. J. Steroid Biochem. Mol. Biol..

[129] Gharib B., Nugon-Baudon L., Lafont R., De Reggi M. (1993). Ecdysteroids, a new pathological marker in man. Biologie Prospective: Compte-rendus du 8è colloque de Pont-à-Mousson.

[130] Schiffer L., Barnard L., Baranowski E.S., Gilligan L.C., Taylor A.E., Arit W., Shackleton C.H.L., Storbeck K.-H. (2019). Human steroid biosynthesis, metabolism and excretion are differentially reflected by serum and urine steroid metabolomes: A comprehensive review. J. Steroid Biochem. Mol. Biol..

[131] Wells J.E., Hylemon P.B. (2000). dentification and characterization of a bile acid 7alpha-dehydroxylation operon in *Clostridium* sp. strain TO-931, a highly active 7alpha-dehydroxylating strain isolated from human feces. Appl. Environ. Microbiol..

[132] Simon P., Koolman J., Koolman J. (1989). Ecdysteroids in vertebrates: Pharmalogical aspects. Ecdysone—from Chemistry to Mode of Action.

[133] Simon P. (1988). Ecdysteroids in the mammalian organism and their detection as a means of diagnosis antihelmintic infections (Ecdysteroide im Säugerorganismus und ihr Nachweis als Möglichkeit der Diagnose helmintischer Infektionen). Ph.D. Thesis.

[134] Bolduc T.M. (2008). Human Urinary Excretion Profiles after Exposure to ecdysterone. Master’s Thesis.

[135] Brandt W. (2003). Pharmakokinetik und Metabolismus des 20-Hydroxyecdysons im Menschen. Ph.D. Thesis.

[136] Dioh W., Del Signore S., Dupont P., Dilda P., Lafont R., Veillet S. (2017). SARA-PK: A Single and Multiple Ascending Oral Doses Study to Assess the Safety and Evaluate the Pharmacokinetics of BIO101 in Healthy Young and Older Volunteers.

[137] Dioh W., Tourette C., Del Signore S., Daudigny L., Balducci C., Dupont P., Dilda P., Agus S., Lafont R., Veillet S. (2021). A phase I, combined study of the safety and pharmacokinetics of BIO101 (20-hydroxyecdysone) in healthy young and elderly adult volunteers after single ascending and multiple ascending oral doses for 14 days.

[138] Thiem B., Kikowska M., Malinski M.P., Kruszka D., Napierala M., Florek E. (2017). Ecdysteroids: Production in plant in vitro cultures. Phytochem. Rev..

[139] Fujimoto Y., Ohyama K., Nomura K., Hyodo R., Takahashi K., Yamada J., Morisaki M. (2000). Biosynthesis of sterols and ecdysteroids in *Ajuga* hairy roots. Lipids.

[140] Chen R., Yang S., Zhang L., Zhou Y.J. (2000). Advanced strategies for the production of natural products in yeast. iScience.

[141] Yan X., Yan Y., Wei W., Wang P., Liu Q., Wei Y., Zhang L., Zhao G., Yue J., Zhou Z. (2014). Production of bioactive ginsenoside compound K in metabolically engineered yeast. Cell Res..

[142] Duport C., Spagnoli R., Degryse E., Pompon D. (1998). Self-sufficient biosynthesis of pregnenolone and progesterone in engineered yeast. Nat. Biotechnol..

[143] Szczebara F.M., Chandelier C., Villeret C., Masurel A., Bourot S., Duport C., Blanchard S., Groisillier A., Testet E., Costaglioli P. (2003). Total biosynthesis of hydrocortisone from a simple carbon source in yeast. Nat. Biotechnol..

[144] Dinan L., Balducci C., Guibout L., Lafont R. (2020). Small-scale analysis of phytoecdysteroids in seeds by HPLC-DAD-MS for the identification and quantification of specific analogues, dereplication and chemotaxonomy. Phytochem. Anal..

[145] Wang J.-L., Ruan D.-C., Cheng Z.-Y., Yang C.-R. (1996). The dynamic variations of 20-hydroxyecdysone in *Cyanotis arachnoidea*. Acta Bot. Yunnanica.

[146] Bandara B.M.R., Jayasinghe L., Karunaratne V., Wanningama G.P., Bokel M., Kraus W. (1989). Ecdysterone from stem of *Diploclisia glaucescens*. Phytochemistry.

[147] Ramazonov N.S.H., Bobaev I.D., Syrov V.N., Sagdullaev S.H., Mamatkhanov A.U. (2016). Chemistry, biology and production technology of phytoecdysteroids. Sci. Technol. Tashkent.

[148] Volodin V.V., Pchelenko L.D., Volodina S.O., Kudyasheva A.G., Shevchenko O.G., Zagorskaya N.P. (2006). Pharmacological estimate of new ecdysteroid-containing substance “Serpisten”. Rastit Resur..

[149] Martnussen I., Volodin V., Volodina S., Uleberg E. (2011). Effect of climate on plant growth and level od adaptogenic compounds in Maral root (*Leuzea carthamoides* Willd., DC), crowned saw-wort (*Serratula coronata* L.) and roseroot (*Rhodiola rosea* L.). Eur. J. Plant. Sci. Biotechnol..

[150] Dinan L. (2001). Phytoecdysteroids: Biological aspects. Phytochem.

[151] Báthori M., Girault J.P., Kalasz H., Mathé I., Dinan L.N., Lafont R. (1999). Complex phytoecdysteroid cocktail of *Silene otites* (Caryophyllaceae). Arch. Insect Biochem. Physiol..

[152] Lobell M., Hendrix M., Hinzen B., Keldenich J., Meier H., Schmeck C., Schohe-Loop R., Wunberg T., Hillisch A. (2006). In silico ADMET traffic lights as a tool for the prioritization of HTS hits. ChemMedChem.

[153] Lipinski C.A., Lombardo F., Dominy B.W., Feeney P.J. (2001). Experimental and computational approaches to estimate solubility and permeability in drug discovery and developments settings. Adv. Drug Deliv. Rev..

[154] Otaka T., Uchiyama M., Okui S., Takemoto T., Hikino H., Ogawa S., Nishimoto N. (1968). Stimulatory effect of insect metamorphosing steroids from *Achyranthes* and *Cyathula* on protein synthesis in mouse liver. Chem. Pharm. Bull..

[155] Issaadi H.M., Csábi J., Hsieh T.J., Gáti T., Tóth G., Hunyadi A. (2019). Side-chain cleaved phytoecdysteroid metabolites as activators of protein kinase B. Bioorg. Chem..

[156] Novikov V.S., Shamarin I.A., Bortnovskii V.N. (1992). A trial of the pharmacological correction of sleep disorders in sailors during a cruise. Voen Med. Zhurnal.

[157] Marina T.F. (1966). Influence of CNS stimulators of plant origin on reflex activity of spinal cord. In: Stimulators of the Central Nervous System. Tomsk.

[158] Mirzaev Y.R., Syrov V.N., Krushev S.A., Iskanderova S.D. (2000). Study of the effects of ecdysten on the sexual function under experimental and clinical conditions. Eksp. Klin. Farm..

[159] Syrov V.N., Khushbaktova Z.A., Komarin A.S., Abidov A.B., Pechenitsina T.V., Aripkhodzhaeva F.A. (2004). Experimental and clinical evaluation of the efficacy of ecdysten in the treatment of hepatitis. Eksp. Klin. Farmakol..

[160] Mosharrof A.H. (1987). Effects of extract from Rhaponticum carthamoides (Willd) Iljin (*Leuzea*) on learning and memory in rats. Acta Physiol. Pharmacol. Bulg..

[161] Opletal L., Sovova M., Dittrich M., Solich P., Dvorák J., Krátký F., Cerovský J., Hofbauer J. (1997). Phytotherapeutic aspects of diseases of the circulatory system. Leuzea carthamoides (WILLD.) DC: The status of research and possible use of the taxon. Ceska Slov. Farm..

[162] Ambrosio G., Wirth D., Joseph J.F., Mazzarino M., de la Torre X., Botrè F., Parr M.K. (2020). How reliable is dietary supplement labelling?—Experiences from the analysis of ecdysterone supplements. J. Pharm. Biomed. Anal..

[163] Isenmann E., Ambrosio G., Joseph J.F., Mazzarino M., de la Torre X., Zimmer P., Kazlauskas R., Goebel C., Botrè F., Diel P. (2019). Ecdysteroids as non-conventional anabolic agent: Performance enhancement by ecdysterone supplementation in humans. Arch. Toxicol..

[164] Parr M.K., Ambrosio G., Wuest B., Mazzarino M., de la Torre X., Sibilia F., Joseph J.F., Diel P., Botré F. (2020). Targeting the administration of ecdysterone in doping control samples. Forensic Toxicol..

[165] Kibrik N.D., Reshetnyak J.A. (1996). Therapeutical approaches to sexual disadaption. Eur. Neuropsychopharmacol..

[166] Saatov Z., Agzamkhodjaeva D.A., Syrov V.N. (1999). Distribution of phytoecdysteroids in plants of Uzbekistan and the possibility of using drugs based on them in nephrological practice. Chem. Nat. Comp..

[167] Osipova S.O., Islamova Z.I., Syrov V.N., Badalova N.S., Khushbaktova Z.A. (2002). Ecdysten in the treatment of giardiasis. Med. Parazitol. (Mosk).

[168] Islamova Z.I., Syrov V.N., Khushbaktova Z.A., Osipova S.O. (2010). The efficacy of ecdystene versus metronidazole in the treatment of lambliasis. Med. Parazitol. (Mosc.).

[169] Makhmudova L.B. (2012). Experience of using ecdisten in the treatment of hymenolepiasis. Med. Parazitol. (Mosc.).

[170] Seidlova-Wuttke D., Wuttke W. (2012). In a placebo-controlled study β-ecdysone (ECD) prevented the development of the metabolic syndrome. Planta Med..

[171] Wuttke M., Seidlova-Wuttke D. (2012). Beta-ecdysone (Ecd) prevents visceral, bone marrow and joint fat accumulation and has positive effects on serum lipids, bone and joint cartilage. Planta Med..

[172] Wuttke W., Seidlova-Wuttke D. (2015). Eine neue Alternative für die Prävention und Therapie postmenopausaler Erkrankungen, insbesondere des metabolischen Syndroms. J. Gynäkol. Endokrinol..

[173] Agus S., Dioh W. (2018). A Double-blind, Placebo Controlled, Randomized INTerventional Clinical Trial (SARA-INT). Clinicaltrials.gov NCT03452488.

[174] Thole S.W. (2018). The Metabolic Syndrome: The Effects of β-ecdysone on Selected Body Parameters and Serum Lipids on the Metabolic Syndrome. Ph.D. Thesis.

[175] Rayas A.L.F. (2019). Effect of phytoecdysterone administration in subjects ith prediabetes. Clinicaltrials.gov identifier NCT03906201.

[176] Agus S., Dioh W. (2020). Testing the Efficacy and Safety of BIO101 for the Prevention of Respiratory Deterioration in COVID-19 Patients (COVA). Clinicaltrials.gov NCT04472728.

[177] Dustmukhamedova D.K.H., Kamilovz A.T. (2020). The characteristic of energy metabolism disorders and its correction in children with celiac disease. Am. J. Med. Medic. Sci..

[178] Kwan P. (2013). Sarcopenia: Neurological point of view. J. Bone Metab..

[179] Drey M., Krieger B., Sieber C.C., Bauer J.M., Hettwer S., Bertsch T., DISARCO Study Group (2014). Motoneuron loss is associated with sarcopenia. J. Am. Med. Dir. Assoc..

[180] Dioh W., Chabane M., Tourette C., Azbekyan A., Morelot-Panzini C., Hajjar L.A., Lins M., Nair G.B., Whitehouse T., Mariani J. (2021). Testing the efficacy and safety of BIO101, for the prevention of respiratory deterioration, in patients with COVID-19 pneumonia (COVA study): A structured summary of a study protocol for a randomised controlled trial. Trials.

[181] Latil M., Camelo S., Veillet S., Lafont R., Dilda P.J. (2021). Developing new drugs that activate the protective arm of the renin-angiotensin system as a potential treatment for repiratory failure in COVID-19 patients. Drug Discov. Today.

[182] WADA Summary of Major Modifications and Explanatory Notes. 2020 prohibited list. https://www.wada-ama.org/sites/default/files/wada_2020_english_summary_of_modifications_.pdf.

